# EEG emotion recognition via multilevel entropy analysis: The power of entropy of entropy on phase dynamics

**DOI:** 10.1371/journal.pone.0329598

**Published:** 2026-10-05

**Authors:** Morteza Jafari Malali, Sepideh Zolfaghari, Raheleh Davoodi

**Affiliations:** 1 Faculty of Electrical Engineering, Shahid Beheshti University, Tehran, Iran; 2 Faculty of Electrical and Computer Engineering, University of Tabriz, Tabriz, Iran; Nanyang Technological University, SINGAPORE

## Abstract

Emotion recognition from EEG signals has become a central task in affective computing, offering non-invasive insight into human emotional states. Due to the nonlinear and nonstationary nature of EEG, entropy-based features have gained prominence as reliable indicators of brain complexity. However, traditional entropy metrics—such as Shannon entropy (ShanEn) and its variants—often lack sensitivity to transient dynamics and multiscale irregularities in the brain’s electrical activity. This study introduces a novel application of Entropy of Entropy (EoE) to EEG phase series (EoE – phase), aiming to enhance the detection of emotional states by capturing second-order variability in signal complexity. Unlike conventional measures, EoE quantifies not just signal unpredictability, but also the fluctuation of that unpredictability over time, offering a more dynamic representation of neural information flow. Using the GAMEEMO dataset, which includes EEG recordings from 28 participants during four emotion-evoking game scenarios (funny, boring, horror, calm), we computed and compared four feature sets: Phase Entropy, EoE – phase, ShanEn, and EoE applied to EEG amplitude time series. These features were classified using six machine learning models, including AdaBoost, SVM, and Random Forest. Our results demonstrate that EoE applied to phase dynamics significantly outperforms other entropy-based features, achieving a classification accuracy of 71.7% and AUC of 0.869 using the RF classifier with mixed PhaseEn and EoE-phase features, while traditional Shannon-based features reached only ~59% accuracy. This study provides the first systematic evaluation of EoE on EEG phase signals for emotion recognition, revealing its strong discriminative power and potential for integration into next-generation brain-computer interfaces.

## Introduction

Understanding and detecting human emotional states from neural activity is a key challenge in affective computing. Among the many modalities used for emotion recognition, Electroencephalography (EEG) has gained particular attention due to its non-invasiveness, fine temporal resolution, and direct measurement of cortical brain activity [1–3]. Emotions are known to modulate electrical patterns in the brain, and decoding these patterns can provide valuable insight for applications such as adaptive interfaces, mental health monitoring, and brain-computer interaction systems [4].

However, EEG signals are inherently nonlinear, nonstationary, and noisy, which limits the effectiveness of traditional linear signal processing methods. As a result, researchers have increasingly turned to nonlinear dynamic analysis and entropy-based methods to characterize EEG complexity [5,6]. From a dynamical systems perspective, emotional states are associated with changes in neural variability and complexity in large-scale cortical networks involved in affective processing. Entropy provides a quantitative framework for capturing these changes in brain dynamics. Entropy measures—quantifying the degree of randomness or unpredictability in a signal—have been extensively used to track changes in brain dynamics associated with different emotional states. These changes reflect emotion-dependent modulation of neural complexity, particularly in large-scale cortical networks involved in affective processing. Classical entropy types like Shannon Entropy (ShanEn) [7] and Sample Entropy (SampEn) [8,9] have been shown to differentiate between high and low arousal, or between positive and negative valence, with reasonable success.

The importance of identifying meaningful EEG features for emotion classification has led to the development and application of more sophisticated entropy models, including Multiscale Entropy [10], Fuzzy Entropy [11,12], Permutation Entropy (PermEn) [13], Differential Entropy (DiffEn) [14], and more recently, Entropy of Entropy (EoE) [15]. These measures attempt to capture better the temporal, spectral, and structural complexity of brain signals. For instance, Multiscale Entropy calculates entropy across multiple time scales, thus revealing coarse and fine-grained dynamics. In contrast, DiffEn uses statistical assumptions about the signal distribution to provide robust metrics across frequency bands [16,17].

In parallel, several benchmark datasets have been developed to support EEG-based emotion analysis. The most prominent among these are the DEAP dataset [1], consisting of 32-channel EEG recorded during the viewing of affective music videos; the SEED dataset [18], which uses 62-channel EEG recordings while participants watch emotional film clips; and the more recent GAMEEMO dataset [19], in which 28 subjects interact with emotion-inducing computer games while wearing a 14-channel wireless EEG headset. GAMEEMO is particularly notable for its more naturalistic and interactive emotion elicitation method, making it an ideal platform for evaluating EEG features under realistic conditions.

Despite significant progress in entropy-based emotion recognition, existing studies have predominantly focused on entropy computed directly from raw EEG amplitude or power spectral density [20,21]. A less explored, yet potentially powerful dimension of EEG is its phase information. The phase of brain oscillations encodes critical timing and synchrony between brain regions, which may be essential in emotional regulation [22]. Phase synchronization reflects temporal coordination between distributed brain regions, which is essential for integrating emotional and cognitive information. Alterations in phase dynamics have been associated with changes in affective state and neural communication efficiency. Capturing the complexity of these phase signals may thus offer richer and more discriminative features than amplitude alone.

This research addresses a key gap in the literature: the lack of systematic exploration of entropy metrics applied to EEG phase dynamics, particularly the EoE, which operates on a two-level structure. This hierarchical formulation enables the capture of second-order variability in signal complexity, providing a more sensitive descriptor of temporal fluctuations in neural dynamics compared to conventional entropy measures. EoE first computes a base entropy over small segments of a signal, then evaluates the entropy of that sequence of entropies. Preliminary studies in other domains, such as cardiac signal analysis [23] and gait stability [24], suggest that EoE offers improved sensitivity to transient dynamical changes. Yet, its use in emotion recognition, especially on the phase component of EEG, remains largely unexplored. When applied to phase signals, EoE can further characterize instability in neural synchronization patterns, which may be directly linked to emotion-specific network reconfiguration.

To fill this gap, our study makes the following key contributions:

- We introduce a hierarchical entropy-based framework to quantify second-order variability in EEG phase dynamics for emotion recognition.
- We conduct a comparative analysis with conventional entropy measures, including ShanEn and PhaseEn, as well as EoE applied to the amplitude of EEG signals in the time domain (EoE – time).
- We use the GAMEEMO dataset as a testbed, enabling a realistic evaluation using game-elicited emotional states (funny, boring, horror, calm).
- We implement a consistent classification pipeline across six classifiers: K-Nearest Neighbors (KNN), Linear Discriminant Analysis (LDA), Support Vector Machine (SVM), Decision Tree (DT), Random Forest (RF), and Adaptive Boosting (AdaBoost), ensuring rigorous benchmarking.
- We demonstrate that phase-based EoE features outperform traditional entropy metrics in both binary and multi-class emotion classification, achieving up to 71.7% accuracy and 0.869 AUC with RF.

These results suggest that second-order entropy analysis of EEG phase dynamics may provide a more sensitive representation of emotion-related neural variability, highlighting its potential utility in affective brain–computer interfaces.

## Related work

A wide range of entropy-based features has been proposed for EEG emotion recognition over the past decade. ShanEn [25,26], rooted in information theory, has been used extensively to capture signal disorder in both time and frequency domains. Spectral entropy [27]—a ShanEn applied to the power spectral density—has proven effective at capturing emotional variations across EEG bands [21]. For example, Alakus et al. reported ~76.9% accuracy in binary emotion recognition using spectral entropy features and BiLSTM models on the GAMEEMO dataset [28].

SampEn, an improvement over Approximate Entropy, calculates the negative log probability that patterns in a signal remain similar when extended by one data point [29]. SampEn is less biased and better suited to short signals. Ji et al. [30] used SampEn with SVM classifiers to distinguish positive and negative emotions with over 80% accuracy. Fuzzy Entropy, which incorporates fuzzy membership functions, has been shown to outperform SampEn in certain contexts due to its higher noise robustness, especially when extended to multiscale versions [31].

PermEn, which evaluates the frequency of ordinal patterns, is both fast and robust to noise, making it ideal for large datasets [32]. Transfer Entropy and Granger Causality have also been used to capture directional information flow between brain regions, showing promise in stress recognition [33].

Recent work has explored novel entropy models. Aung et al. introduced Multivariate Multiscale Modified Distribution Entropy (MM-mDistEn), which jointly models spatial and temporal entropy, achieving over 95% accuracy on GAMEEMO [34]. Tonoyan et al. proposed Multiscale Rényi Entropy for emotion classification using mid-frontal EEG channels, showing strong discrimination across five emotion classes [35].

Yet, these approaches typically rely on amplitude-based features. The phase domain, although rich in timing and synchronization cues, has been underutilized. Jafari et al. demonstrated that EoE of phase signals could distinguish between cognitive states with over 98% accuracy, suggesting strong potential [23]. To date, however, no published study has systematically examined EoE applied to EEG phase for emotion recognition. Our work is the first to do so, bridging nonlinear dynamic theory with applied affective computing.

By contextualizing EoE within the landscape of EEG entropy research, our study establishes a novel yet theoretically grounded approach that enhances sensitivity to emotional neural dynamics and supports future integration into real-time emotion-aware systems.

## Materials and methods

The GAMEEMO dataset was used in this study, which contains EEG signals recorded with 14 electrodes while participants played computer games. These signals were processed to distinguish four emotional states. Feature extraction was performed using different entropy-based methods: ShanEn, PhaseEn, and EoE – time, EoE – phase. For each type of feature, classifiers were employed to distinguish emotions. Additionally, channels showing significant differences across experimental conditions were identified using statistical analysis.

### Dataset: GAMEEMO

This study utilizes the GAMEEMO dataset introduced by Alakus et al. [19], a game-based EEG emotion dataset comprising recordings from 28 healthy participants aged 20–27. Each participant engaged in four 5-minute computer games designed to elicit specific emotional states: *funny*, *boring*, *horror*, and *calm*. After each session, participants rated their emotional experience using the Self-Assessment Manikin, capturing self-reported arousal and valence. EEG signals were collected using a 14-channel Emotiv Epoc+ wireless headset at a sampling rate of 128 Hz. The device provides 16 electrode locations (AF3, F7, F3, FC5, T7, P7, O1, O2, P8, T8, FC6, F4, F8, AF4, P3, and P4), of which 14 are active EEG channels and two (P3 and P4) serve as reference electrodes. Only the 14 active channels were used for feature extraction and subsequent analysis, while the reference electrodes were excluded. GAMEEMO differs from datasets like DEAP [1] and SEED [18] in its interactive, task-driven elicitation, which may induce more naturalistic emotional states. Compared to DEAP (music videos) and SEED (film clips), GAMEEMO’s game stimuli produce stronger, task-induced physiological engagement. Its major advantages include: (1) balanced four-class emotion design; (2) preprocessed, artifact-reduced signal availability; (3) strong signal clarity due to active engagement. Limitations include a limited number of EEG channels and the absence of a neutral baseline. The overall processing pipeline of the proposed method is summarized in Fig 1. According to the original dataset publication [19], the EEG signals were downsampled to 128 Hz and recorded within an effective bandwidth of 0.16–43 Hz using the built-in high- and low-frequency filters of the Emotiv EPOC+ device. Furthermore, the released preprocessed recordings were processed using the device’s built-in 5th-order sinc filter, which suppresses movement-related and external artifacts while preserving the lower-frequency EEG components. Therefore, no additional preprocessing, filtering, or artifact rejection was performed in the present study before feature extraction.

**Fig 1:**
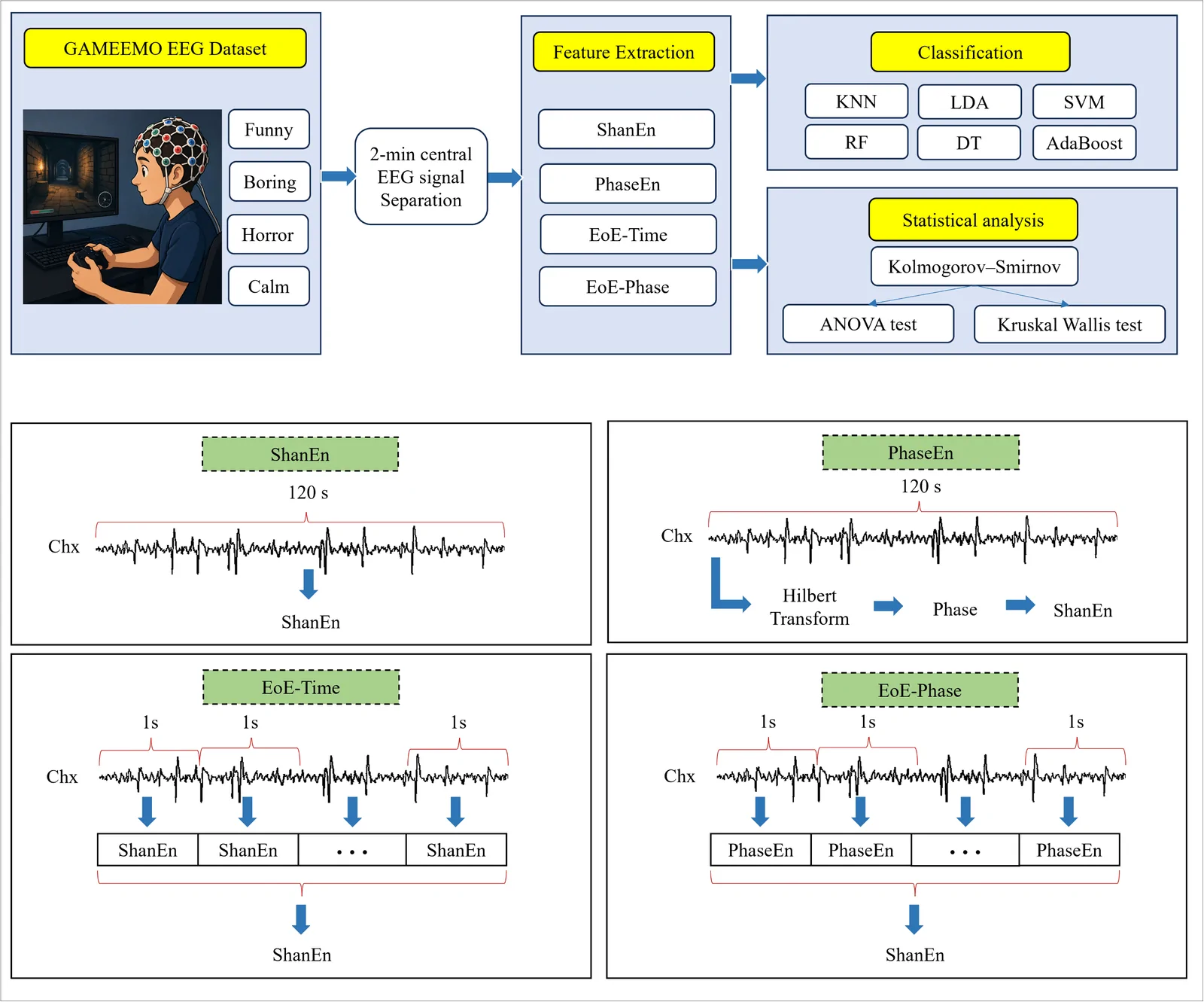
The overall block diagram of the proposed approach. Two-minute EEG segments from the GAMEEMO dataset are used to extract four types of entropy features. For each feature type, statistical analysis was performed to identify significant channels, and classifiers were trained to recognize emotions.

### Feature extraction

To extract features from EEG signals, four entropy-based feature extraction techniques (ShanEn, PhaseEn, EoE – time, and EoE – phase) were employed. These methods were used on EEG segments, each consisting of a central 120 seconds of recordings across 14 channels. All features were computed independently for each EEG channel.

#### ShanEn.

ShanEn was used to quantify the randomness of the EEG amplitude distribution. For a discrete probability distribution

$P=\{P_{\text{1}},P_{\text{2}},\ldots ,P_{n}\;\}$, ShanEn is defined as:


$$\begin{array}{c} H(X)=-\sum_{i=\text{1}}^{n}p_{i}{\text{log}}_{\text{2}}p_{i} \end{array}$$ (1)

Where $p_{i}$ is the normalized probability of occurrences in bin i, ShanEn was computed independently for each channel over the entire EEG signal.

#### PhaseEn.

PhaseEn measures the irregularity of the instantaneous phase of the EEG signal. First, the analytic signal was obtained using the Hilbert transform:


$$\begin{array}{c} \phi (t)=\text{arg}\left\{x(t)+j\cdot HilTr\left[x(t)\right]\right\} \end{array}$$ (2)

Where HilTr[x(t)] is the Hilbert transform of the EEG signal x(t), the phase range [−π, π] was divided into N=12 equal-width bins, and the probability distribution of phase values was obtained from the resulting histogram. ShanEn was then calculated from this distribution to obtain the PhaseEn value for each EEG channel.

#### EoE (Time domain and phase domain).

EoE was used to capture higher-order variability in signal complexity. For calculating the EoE, the length of a 120-second EEG signal was divided into 120 non-overlapping segments. An entropy measure was computed for each segment, forming an entropy sequence.

- Time Domain: ShanEn was applied to each segment $w_{k}$ and created the entropy sequence $\left\{H\left(w_{\text{1}}\right),\;H\left(w_{\text{2}}\right),\ldots ,H\left(w_{\text{1}\text{2}\text{0}}\right)\right\}$
- Phase Domain: PhaseEn was computed for each segment $\Phi_{k}$, obtaining the sequence $\left\{H\left(\Phi_{\text{1}}\right),\;H\left(\Phi_{\text{2}}\right),\ldots ,H\left(\Phi_{\text{1}\text{2}\text{0}}\right)\right\}$

The final EoE–time and EoE–phase values were calculated by constructing a histogram of the entropy sequence using M = 5 bins and computing the ShanEn of this distribution. To evaluate the robustness of the selected EoE parameters, a sensitivity analysis was performed by varying the segment duration, sequence length (N), and histogram bin number (M). As summarized in Supplementary S1 Table, the combination including M = 5 consistently yielded stable results and a higher number of statistically significant EEG channels than the tested alternative (M = 8) in most configurations. Therefore, M = 5 was adopted for all subsequent analyses.

#### Parameter selection and sensitivity analysis.

The parameters N = 12 for PhaseEn, M = 5 for EoE histogram binning, and the 1-second segmentation length were selected based on preliminary empirical experiments. Since these parameters are applied sequentially within a unified signal processing pipeline (segmentation → Hilbert transform → phase extraction → PhaseEn → EoE), their impact was evaluated at the pipeline level rather than in isolation. Multiple candidate values for each parameter were tested, and the resulting number of statistically significant channels (p < 0.05) for EoE–phase was compared as an indicator of feature robustness and discriminative power. The detailed sensitivity analysis is reported in S1 Table.

### Statistical analysis

Normality was assessed using the Kolmogorov–Smirnov test (α=0.05). If distributions met normality assumptions, a one-way analysis of variance (ANOVA) was used to compare the emotional groups across channels. Otherwise, the non-parametric Kruskal–Wallis test was performed. When statistically significant differences were observed, post hoc pairwise comparisons were conducted. Tukey’s HSD test was used following ANOVA, while Dunn’s test was applied following the Kruskal-Wallis test. Bonferroni correction was employed to control for multiple comparisons. The adjusted p-values were calculated as:


$$\begin{array}{c} P_{adjusted}=P_{raw}\times m \end{array}$$ (3)

Where $m=\frac{k(k-\text{1})}{\text{2}}$ represents the number of pairwise comparisons, and k=4 denotes the number of emotional groups [36].

### Classification framework

To evaluate the discriminative capability of the proposed EoE-phase features, a supervised machine learning framework was implemented as a validation step rather than the primary objective of this study.

Each sample corresponds to one subject–emotion pair, resulting in a feature matrix of size 112 × 14 (28 subjects × 4 emotional states). No temporal averaging was performed before feature extraction; entropy features were computed directly from the raw EEG segment of each channel. Since the publicly available preprocessed EEG recordings were directly used in this study, no additional band-pass filtering, artifact removal, or frequency-band decomposition was performed before feature extraction. Therefore, the features reflect the full broadband EEG spectrum without any averaging or selection across frequency bands.

To ensure unbiased evaluation and avoid data leakage, a subject-independent cross-validation strategy was adopted. Specifically, all samples belonging to a given subject were kept exclusively in either training or testing sets. A 10-repetition, 5-fold stratified subject-wise cross-validation was used to ensure robustness across different partitions. This subject-wise partitioning strategy prevents information leakage and provides a more realistic estimate of the model’s generalization performance to previously unseen participants.

Six classifiers were employed: KNN, LDA, SVM, DT, RF, and AdaBoost. These classifiers were selected to represent both linear and nonlinear decision boundaries. The following hyperparameters were used for each classifier: KNN with k = 5 and Euclidean distance; LDA with default parameters (linear discriminant, no regularization); SVM with a linear kernel, data standardization, and one-vs-one coding using fitcecoc; Decision Tree with default parameters (Gini impurity, no pruning); AdaBoost implemented via AdaBoostM2 (fitcensemble, ‘Method’, ‘AdaBoostM2’) with 100 learning cycles. The Random Forest classifier was implemented using MATLAB’s fitcensemble function with the Bagging method. The default MATLAB hyperparameters were used to ensure a fair comparison among classifiers. Specifically, the ensemble consisted of 100 decision trees with classification trees as the base learners, a minimum leaf size of 1, a minimum parent size of 2, and the default number of randomly selected predictors at each split (equal to the square root of the total number of predictors). These implementation details have been added to improve reproducibility and facilitate future comparisons with alternative classifiers.

Performance was evaluated using Accuracy, Macro-averaged F1-score, Area Under the ROC Curve (AUC-ROC), and Area Under the Precision–Recall Curve (AUC-PR). All models were implemented in MATLAB R2022a. Confusion matrices and ROC/PR curves were generated based on aggregated predictions across all folds within each repetition. The numerical results reported in Table 1 are the mean ± standard deviation over 10 independent repetitions, following the procedure described in Algorithm 1. The confusion matrices and ROC/PR curves presented in the figures are selected from the repetition with the highest mean accuracy for visualization purposes only; this visual selection does not influence the reported mean values, standard deviations, or any statistical comparisons (e.g., paired t-tests or Friedman tests) conducted in this study. To avoid any ambiguity, we have explicitly clarified that all numerical evaluations and statistical conclusions are based on the averaged results across all repetitions.

**Table 1:** Classification accuracy, F1-score, and AUC results for all feature sets and classifiers (mean± standard deviation over 10 runs).

Feature Set	Classifier	Accuracy	F1-score	AUC-ROC	AUC-PR
**PhaseEn**	KNN	0.216 ± 0.024	0.199 ± 0.020	0.516 ± 0.018	0.224 ± 0.008
	LDA	0.288 ± 0.034	0.272 ± 0.037	0.581 ± 0.019	0.288 ± 0.012
	SVM	0.328 ± 0.034	0.313 ± 0.035	0.604 ± 0.019	0.299 ± 0.019
	Tree	0.428 ± 0.038	0.413 ± 0.038	0.649 ± 0.028	0.276 ± 0.011
	RF	0.636 ± 0.032	0.626 ± 0.035	0.828 ± 0.023	0.550 ± 0.028
	AdaBoost	0.600 ± 0.038	0.592 ± 0.040	0.788 ± 0.031	0.514 ± 0.034
**EoE – phase**	KNN	0.310 ± 0.024	0.280 ± 0.026	0.661 ± 0.015	0.240 ± 0.012
	LDA	0.380 ± 0.025	0.355 ± 0.026	0.646 ± 0.016	0.293 ± 0.011
	SVM	0.390 ± 0.030	0.372 ± 0.030	0.646 ± 0.017	0.277 ± 0.016
	Tree	0.506 ± 0.036	0.478 ± 0.036	0.713 ± 0.030	0.246 ± 0.027
	RF	0.684 ± 0.037	0.668 ± 0.041	0.867 ± 0.021	0.442 ± 0.017
	AdaBoost	0.682 ± 0.021	0.669 ± 0.026	0.874 ± 0.019	0.461 ± 0.022
**ShanEn**	KNN	0.288 ± 0.010	0.260 ± 0.015	0.625 ± 0.012	0.213 ± 0.014
	LDA	0.298 ± 0.026	0.276 ± 0.030	0.554 ± 0.022	0.256 ± 0.010
	SVM	0.288 ± 0.035	0.259 ± 0.038	0.577 ± 0.022	0.257 ± 0.011
	Tree	0.381 ± 0.053	0.359 ± 0.055	0.624 ± 0.043	0.235 ± 0.020
	RF	0.579 ± 0.028	0.554 ± 0.027	0.778 ± 0.013	0.382 ± 0.018
	AdaBoost	0.588 ± 0.049	0.572 ± 0.043	0.794 ± 0.015	0.390 ± 0.020
**EoE – time**	KNN	0.324 ± 0.029	0.289 ± 0.029	0.544 ± 0.021	0.242 ± 0.013
	LDA	0.296 ± 0.048	0.282 ± 0.049	0.557 ± 0.031	0.284 ± 0.025
	SVM	0.300 ± 0.030	0.284 ± 0.029	0.552 ± 0.018	0.267 ± 0.012
	Tree	0.291 ± 0.032	0.278 ± 0.035	0.532 ± 0.018	0.224 ± 0.010
	RF	0.355 ± 0.037	0.341 ± 0.040	0.610 ± 0.023	0.317 ± 0.018
	AdaBoost	0.319 ± 0.042	0.311 ± 0.042	0.575 ± 0.030	0.301 ± 0.015
**Mixed – PhaseEn + EoE – phase**	KNN	0.339 ± 0.018	0.310 ± 0.021	0.674 ± 0.015	0.246 ± 0.021
	LDA	0.374 ± 0.028	0.352 ± 0.027	0.675 ± 0.011	0.297 ± 0.010
	SVM	0.421 ± 0.045	0.400 ± 0.044	0.701 ± 0.016	0.298 ± 0.014
	Tree	0.528 ± 0.033	0.497 ± 0.027	0.744 ± 0.029	0.263 ± 0.019
	RF	0.717 ± 0.033	0.701 ± 0.035	0.869 ± 0.017	0.463 ± 0.019
	AdaBoost	0.687 ± 0.023	0.668 ± 0.027	0.852 ± 0.017	0.450 ± 0.021

Algorithm 1. Classification pipeline.

Input: EEG signals (28 subjects × 4 emotions × 14 channels)

Output: Accuracy, F1, AUC-ROC, AUC-PR

1. For each subject-emotion pair:

 - Segment 120s EEG into 1s windows

 - Extract PhaseEn and EoE-phase features per channel

 - Form a feature vector of length 14

2. Create feature matrix X (112 × 14), label vector y (4 classes)

3. For repetition = 1–10:

 - Partition subjects into 5 folds (subject-wise stratification)

 - For each fold:

  - Train classifiers on 4 folds with specified hyperparameters

  - Test on held-out fold

  - Store predictions

 - Compute macro-averaged metrics across folds

4. Report mean ± std across 10 repetitions

## Results

This section presents the experimental findings of our multilevel entropy-based EEG emotion recognition framework. The results are structured into two main parts: (1) entropy feature distribution and statistical validation, and (2) classification performance using multiple classifiers.

### Entropy feature distribution and statistical significance

To assess the discriminative power of each feature, we computed and visualized average entropy values across all 14 EEG channels and emotional classes. To determine whether parametric or non-parametric tests were appropriate, the normality of feature distributions was first assessed using the Kolmogorov–Smirnov test. A summary of normality outcomes, the corresponding statistical test applied (ANOVA with Tukey post-hoc for normal data; Kruskal-Wallis with Dunn post-hoc for non-normal data), and the resulting p-values for each channel and feature is provided in Supplementary S2 Table.

#### PhaseEn.

As shown in Fig 2, PhaseEn exhibits relatively the same results in all classes of emotions. The Kruskal–Wallis test results in Fig 3 confirm statistical significance in channel F4 (p < 0.05), suggesting a limited and channel-specific role (primarily F4) in distinguishing emotional responses. Overall, PhaseEn exhibits weak global discriminative capability across channels, indicating limited sensitivity to emotion-related phase dynamics compared to higher-order measures.

**Fig 2:**
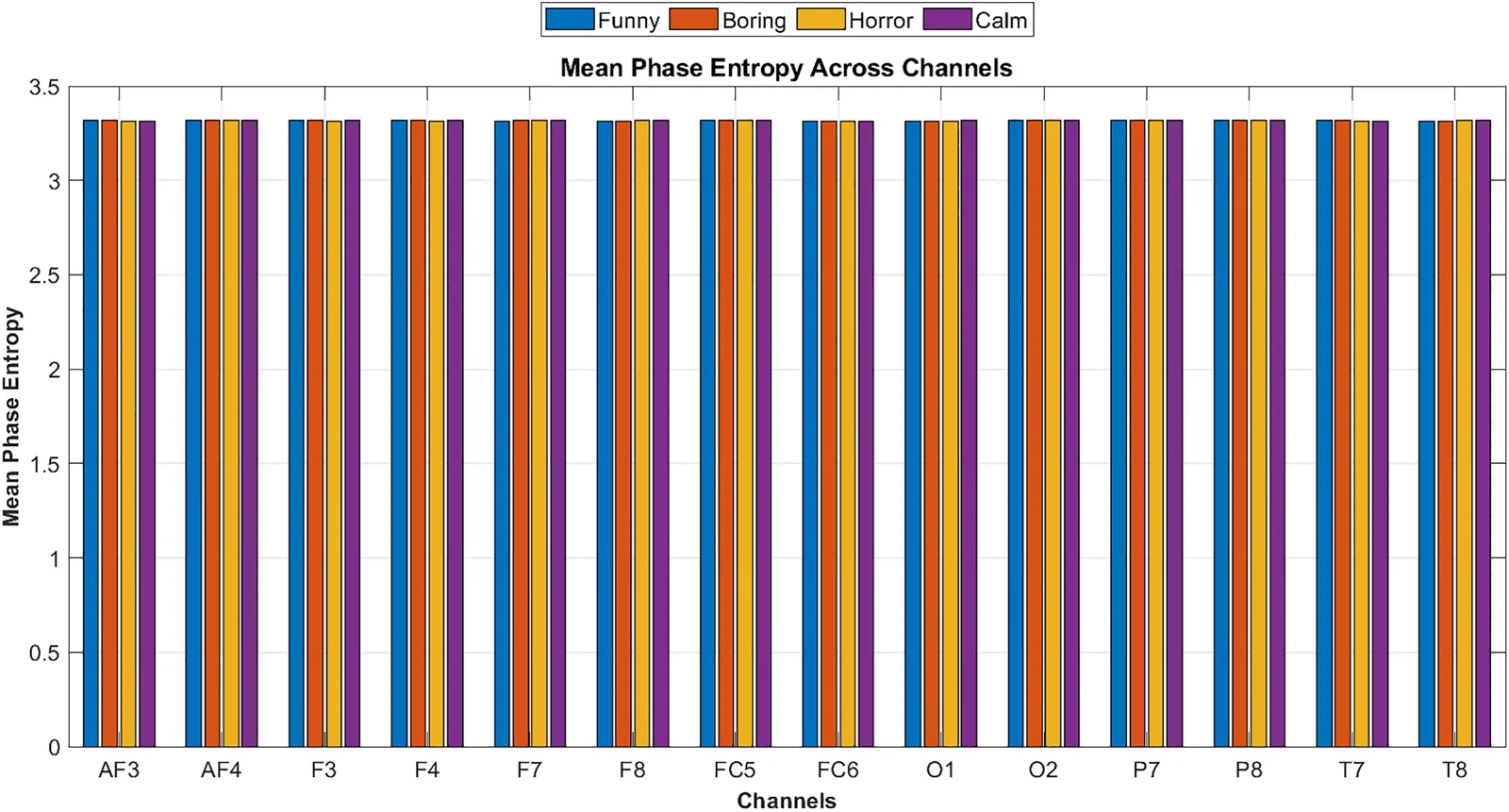
Mean PhaseEn values across all EEG channels for each emotion.

**Fig 3:**
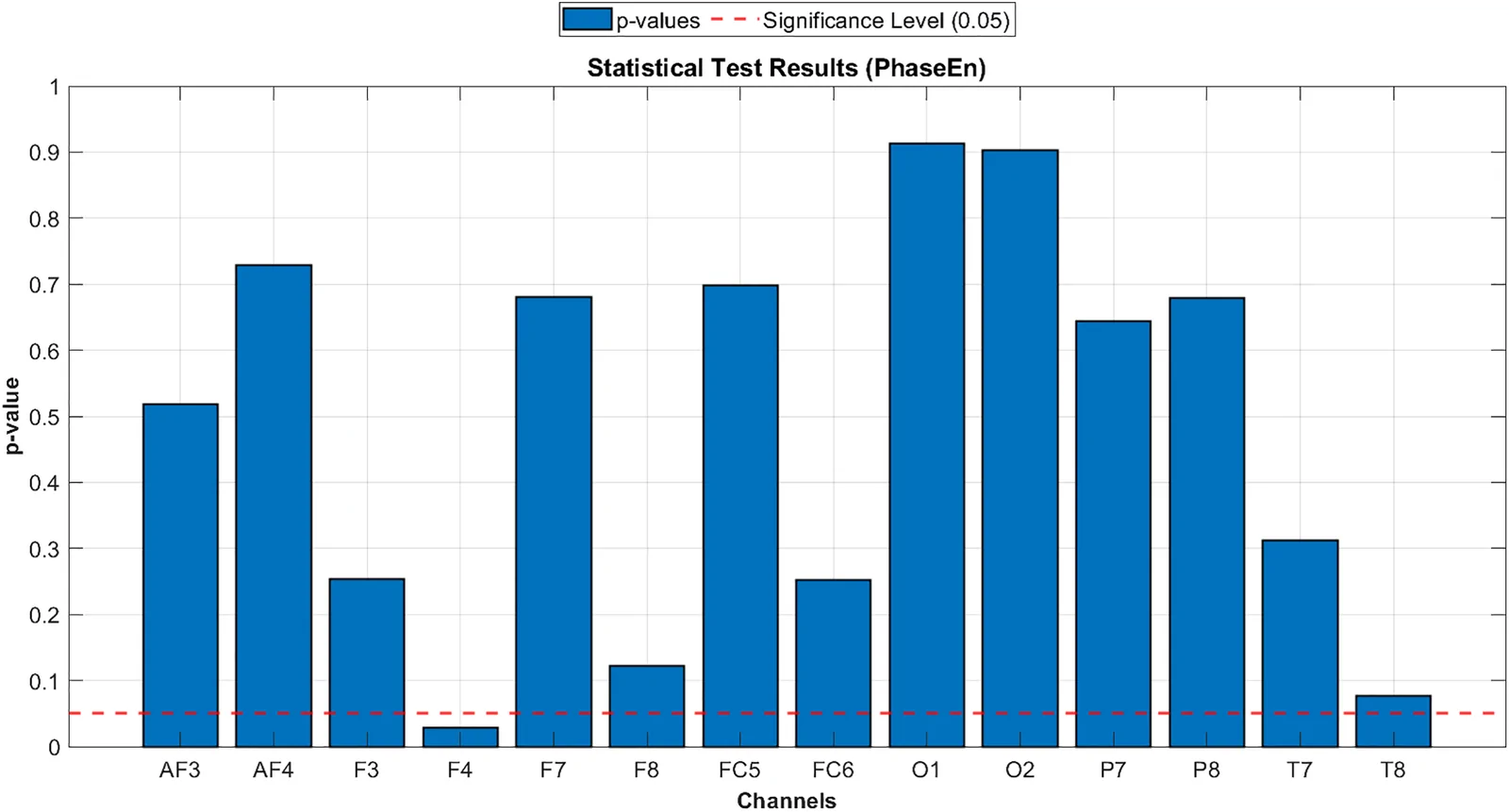
P-values from statistical tests across EEG channels for PhaseEn. F4 channel below the red threshold line (p < 0.05) indicates significant emotional differentiation.

#### EoE – phase.

The sequence of Phase Entropy values was used to compute second-order entropy (EoE), thereby capturing temporal fluctuations in neural complexity. Fig 4 reveals that EoE – phase values for the “boring” and “horror” conditions are elevated across temporal and frontal channels, with maximal values in T7 and T8. The corresponding p-values in Fig 5 highlight significant class differences in T7, T8, O2, P8, and AF4 (all p < 0.05), confirming the utility of EoE – phase in emotion differentiation.

**Fig 4:**
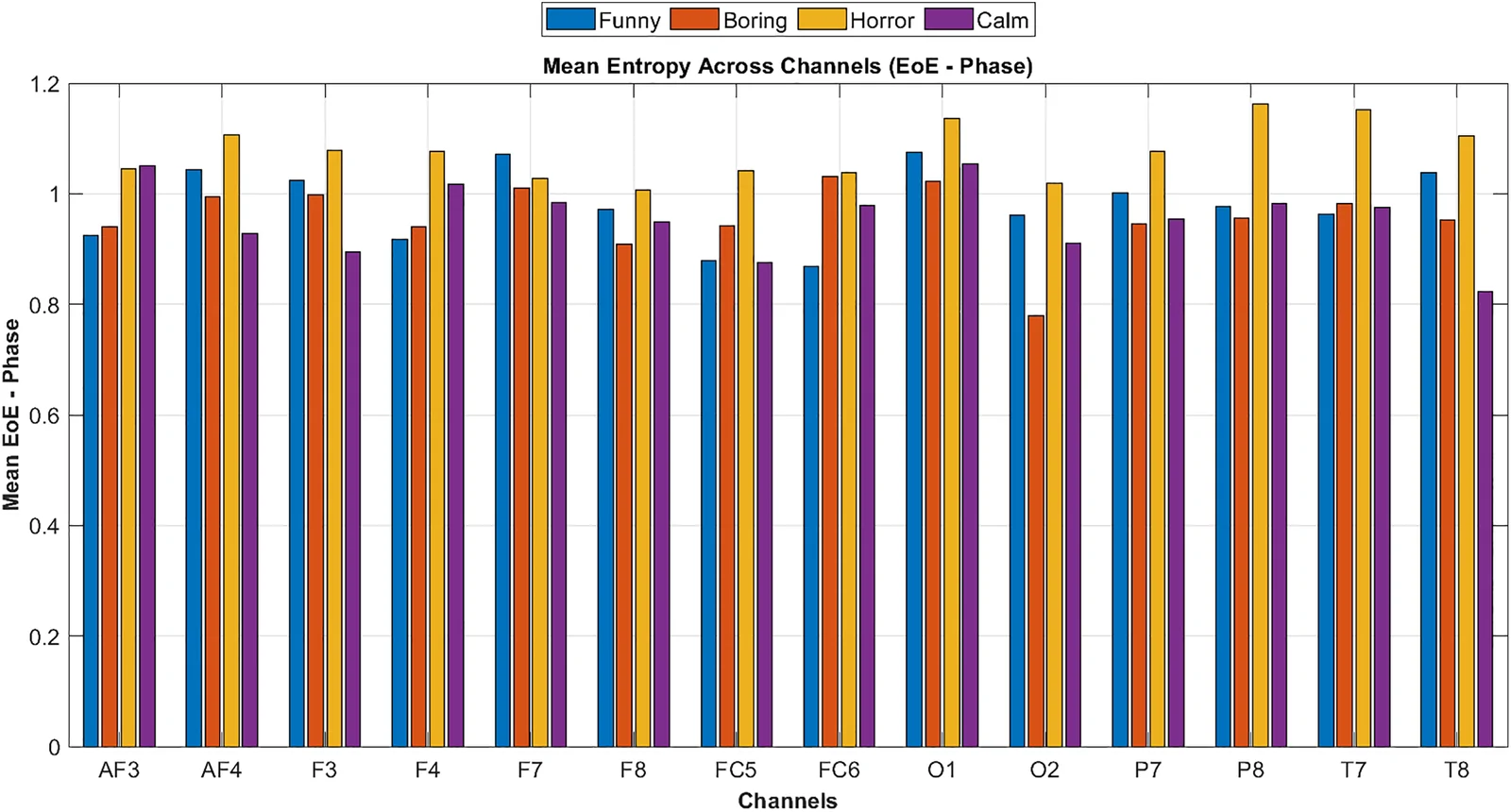
Average EoE – phase values across EEG channels for four emotional states.

**Fig 5:**
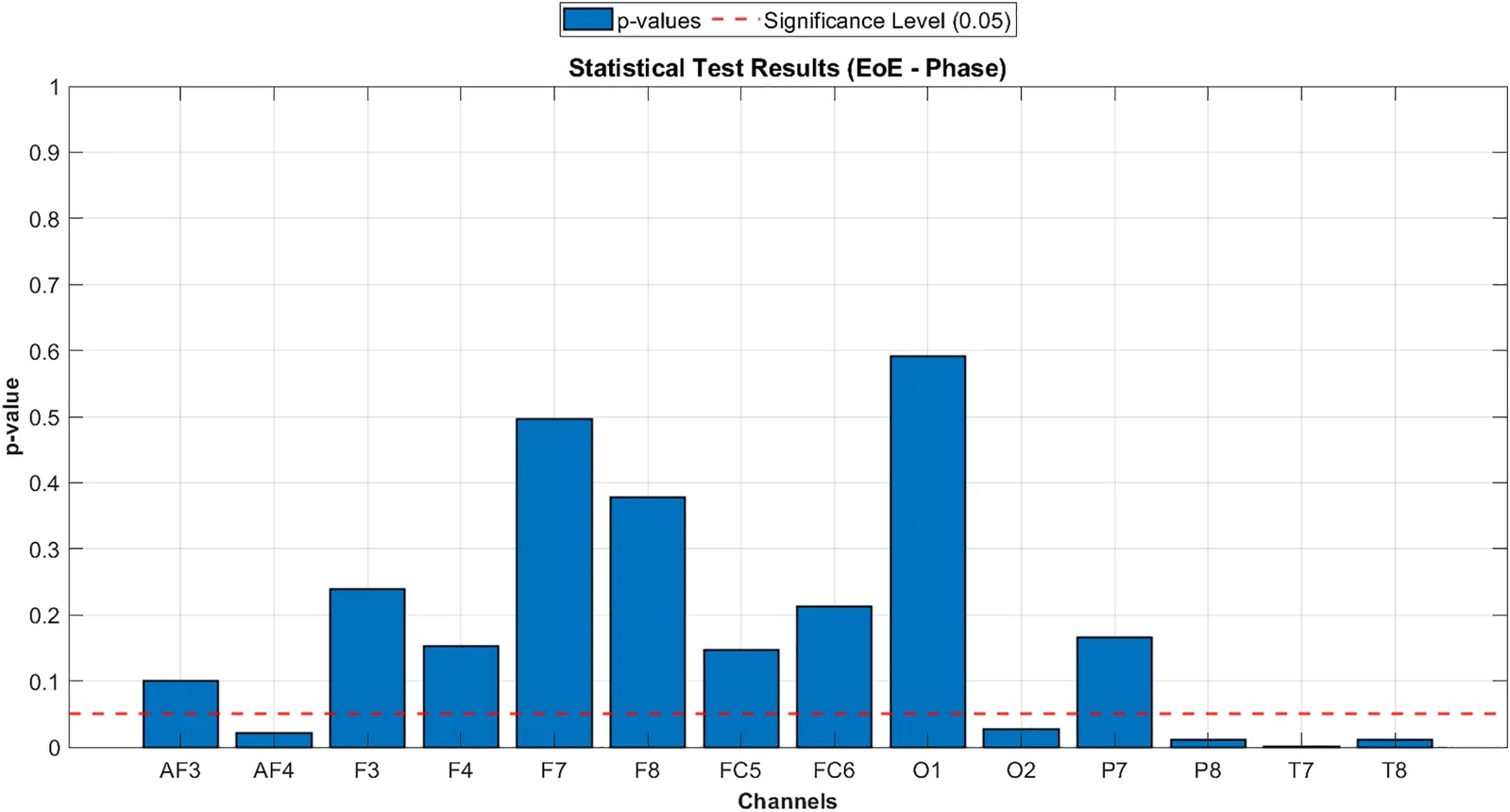
P-values for EoE – phase across EEG channels. Channels such as T7, T8, O2, P8, and AF4 exhibit high discriminative significance.

Fig 6 depicts a topographical representation of significant EEG channels. The left panel shows the position of 14 electrodes and references, whereas the right panel highlights channels with high discriminative power across emotional states. This figure emphasizes the role of frontal, temporal, and occipital regions in emotion recognition utilizing EoE – phase features.

**Fig 6:**
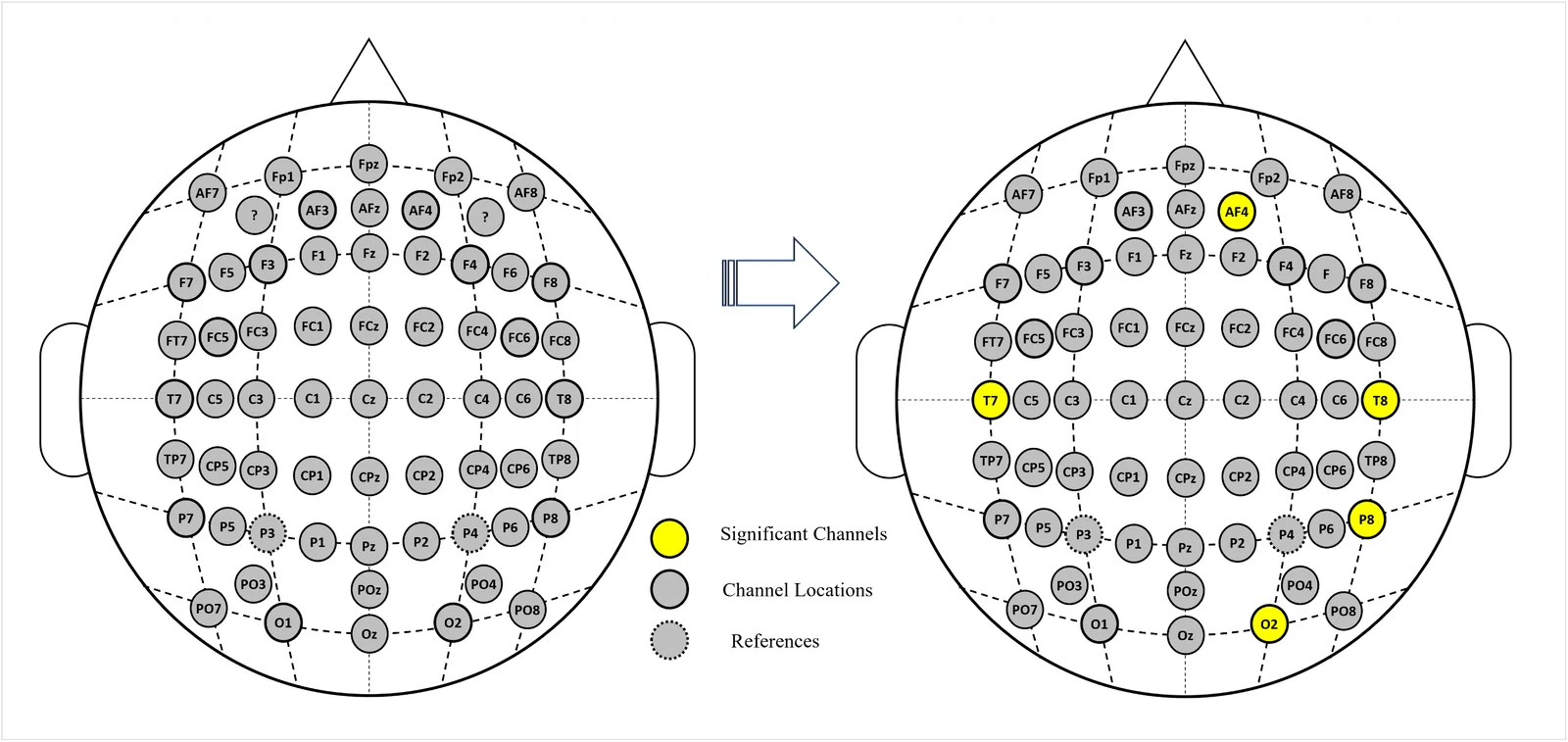
Significant channels according to EoE – phase feature. Channels such as T7, T8, O2, P8, and AF4 exhibit high discriminative significance.

To illustrate how the EoE‑phase feature is derived, a representative phaseEn sequence is shown in Fig 7. The figure presents the temporal evolution of phase entropy values computed from consecutive EEG segments for a representative subject at channel T8 under four emotional states. Each curve corresponds to the entropy sequence obtained from the phase time series. These sequences are subsequently used to construct a histogram distribution, from which the EoE – phase feature is calculated.

**Fig 7:**
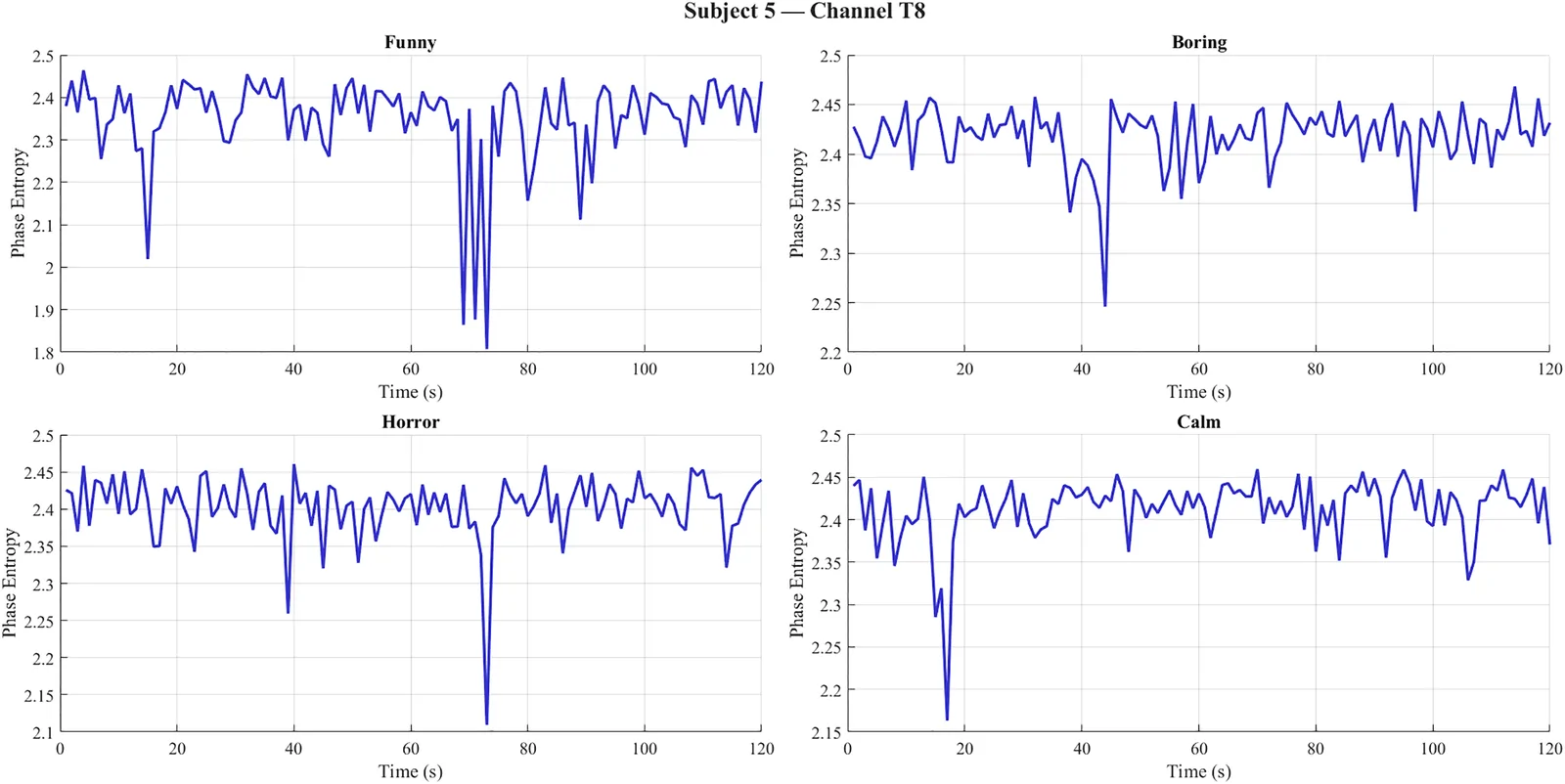
Representative phaseEn sequences for a sample subject at channel T8 under four emotional states (Funny, Boring, Horror, and Calm). Each plot shows the phase entropy values computed from consecutive EEG segments (120 segments), which are used to derive the EoE – phase feature.

#### ShanEn and EoE – time.

The average ShanEn across channels Fig 8 is more evenly distributed but generally elevated in frontal and temporal regions during “boring” state. However, statistical tests in Fig 9 show no channel-level significance, indicating lower discriminability.

**Fig 8:**
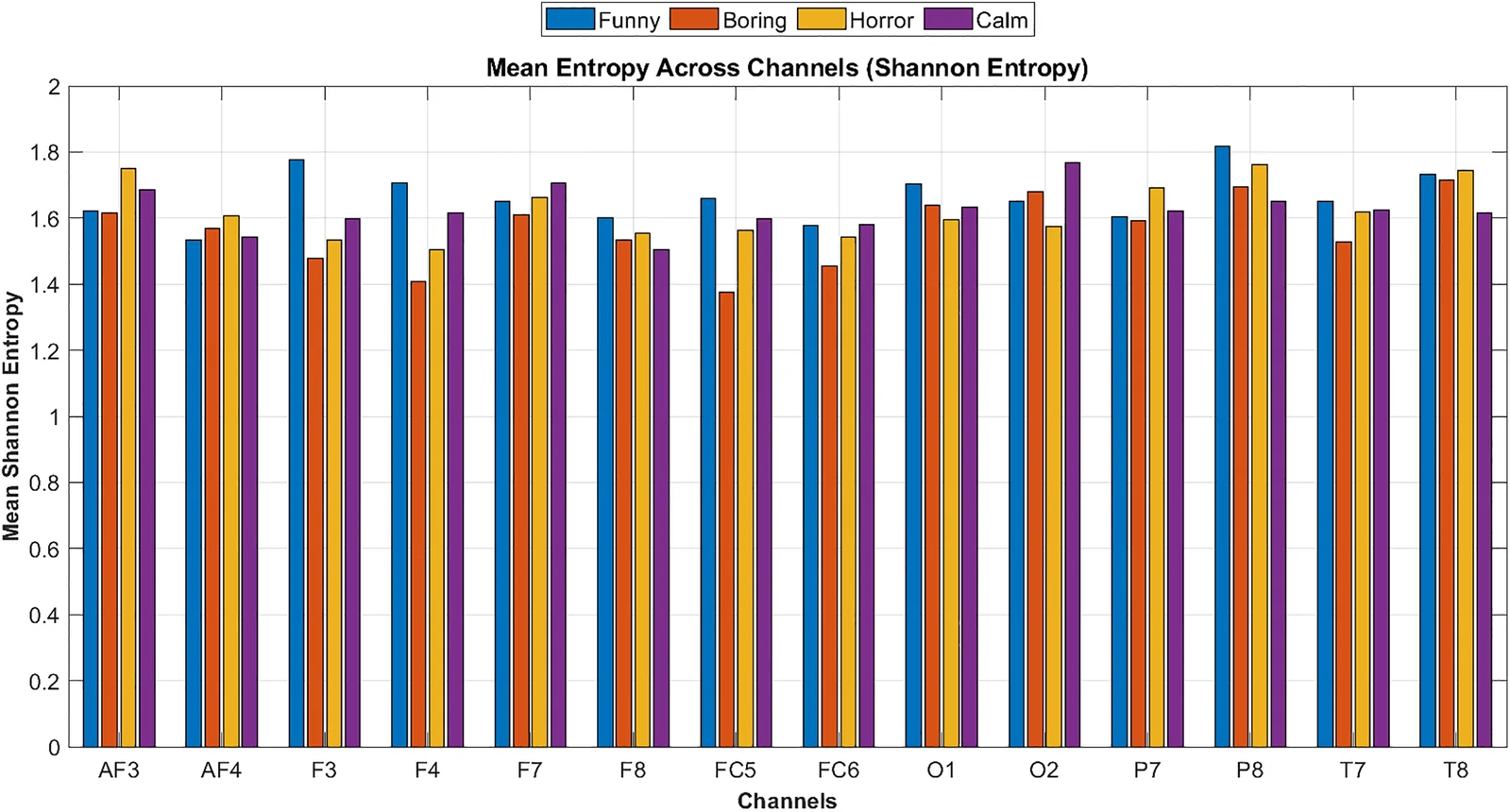
Mean ShanEn across EEG channels.

**Fig 9:**
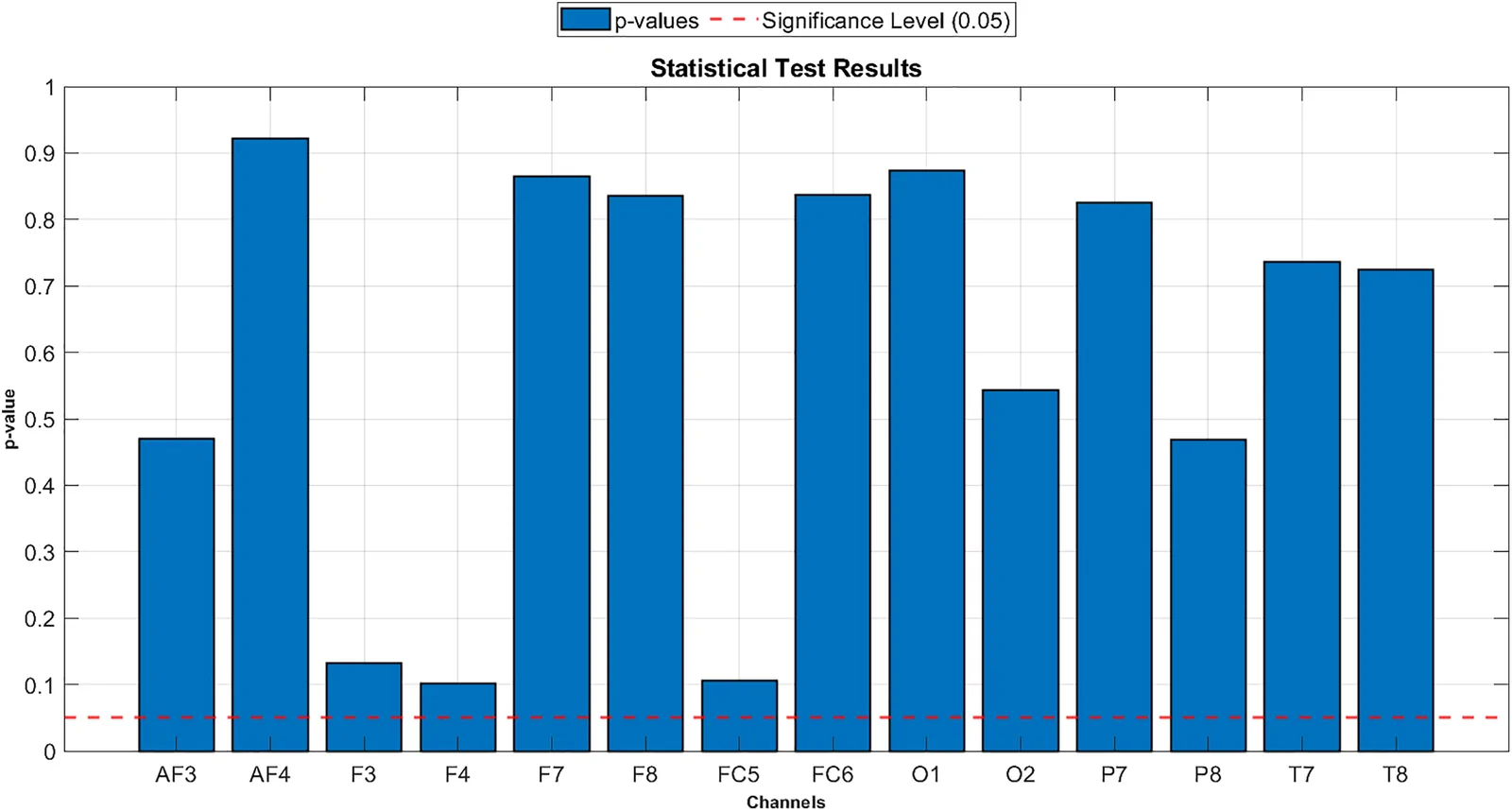
P-values across channels for ShanEn.

Similarly, EoE – time is presented in Fig 10. Though more consistent than ShanEn alone, it has moderate discriminative power. The p-values in Fig 11 show some significance in F7 and P8 electrodes, but not as consistently as EoE – phase features.

**Fig 10:**
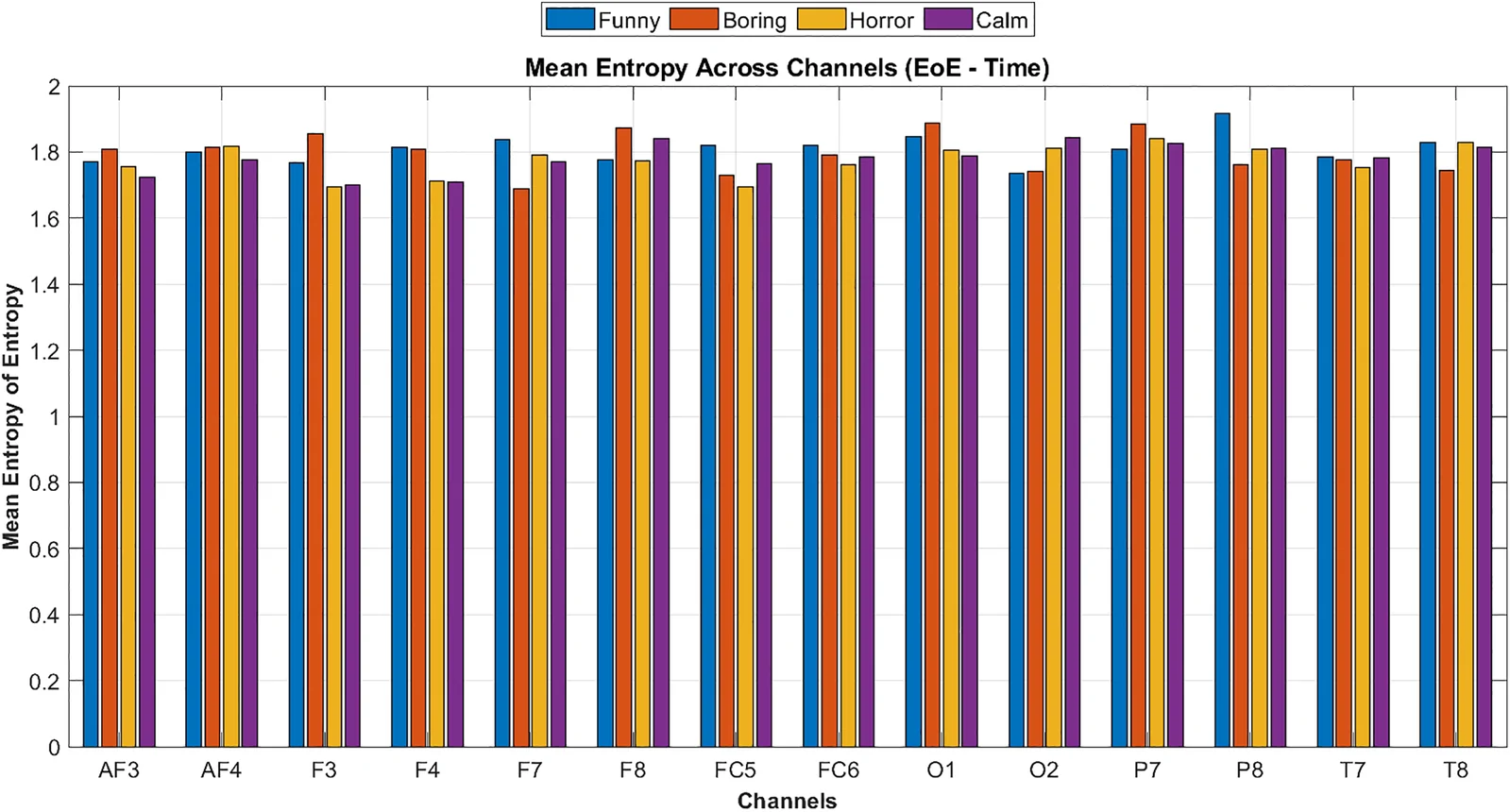
Mean EoE – time across EEG channels.

**Fig 11:**
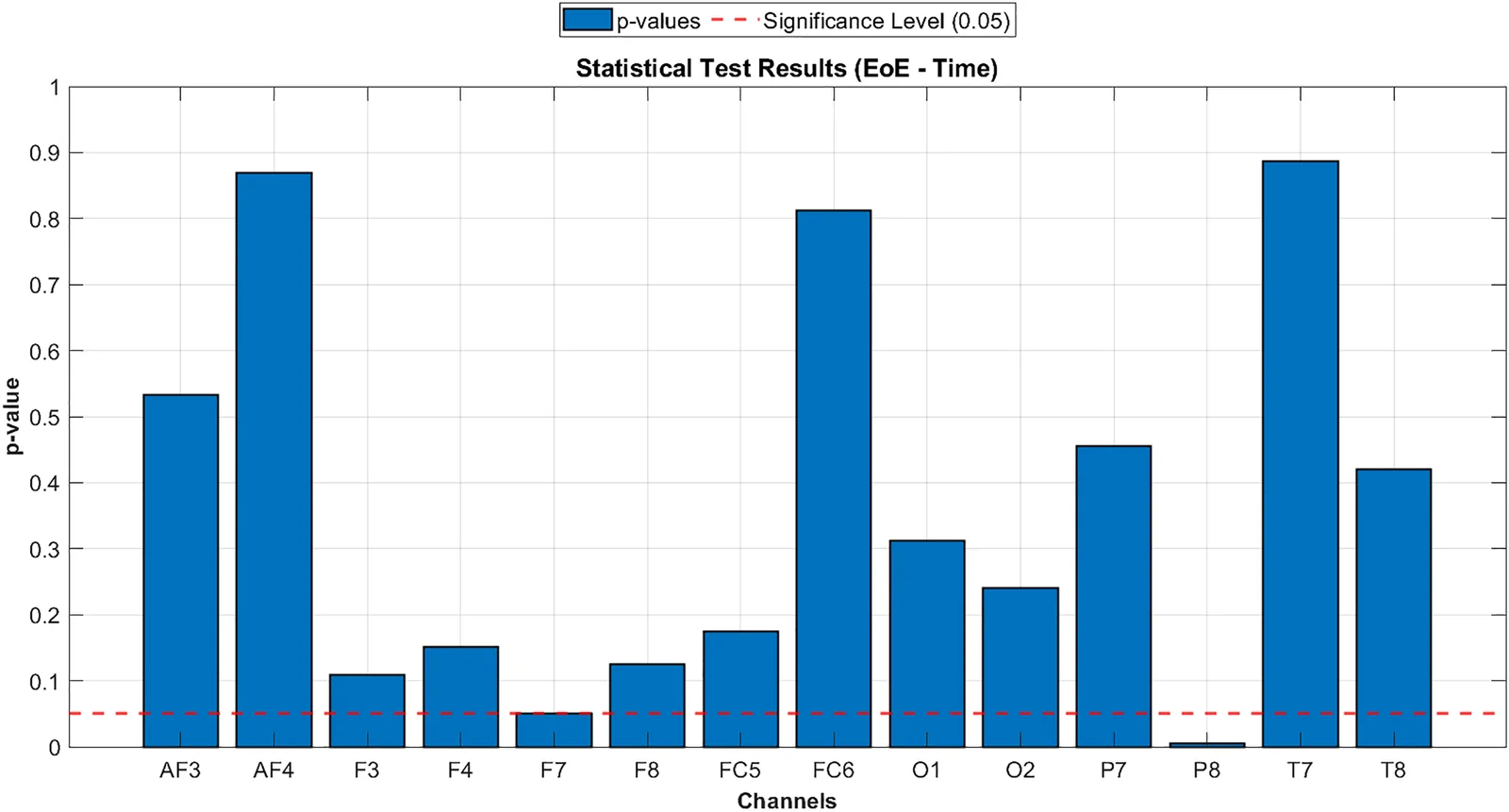
P-values for EoE – time across EEG channels. Moderate significance was observed across two central and frontal electrodes.

### Classification performance

Four feature sets were evaluated: PhaseEn, EoE–phase, ShanEn, and EoE–time. Each feature set was used to train six classifiers (KNN, LDA, SVM, DT, RF, and AdaBoost), and the results are summarized in Table 1. Among the single-feature configurations, the RF classifier trained on EoE–phase features achieved the highest performance, with an average accuracy of 68.4%, an F1-score of 0.67, and an AUC-ROC of 0.87. The corresponding confusion matrix (Fig 12) and ROC curve (Fig 13) indicate particularly strong classification for the “Horror” class, whereas higher confusion is observed for the “Funny” class. Comparable but lower performance was obtained using PhaseEn features alone (Fig 14 and Fig 15), yielding an accuracy of 63.6%, an F1-score of 0.626, and an AUC-ROC of 0.828.

**Fig 12:**
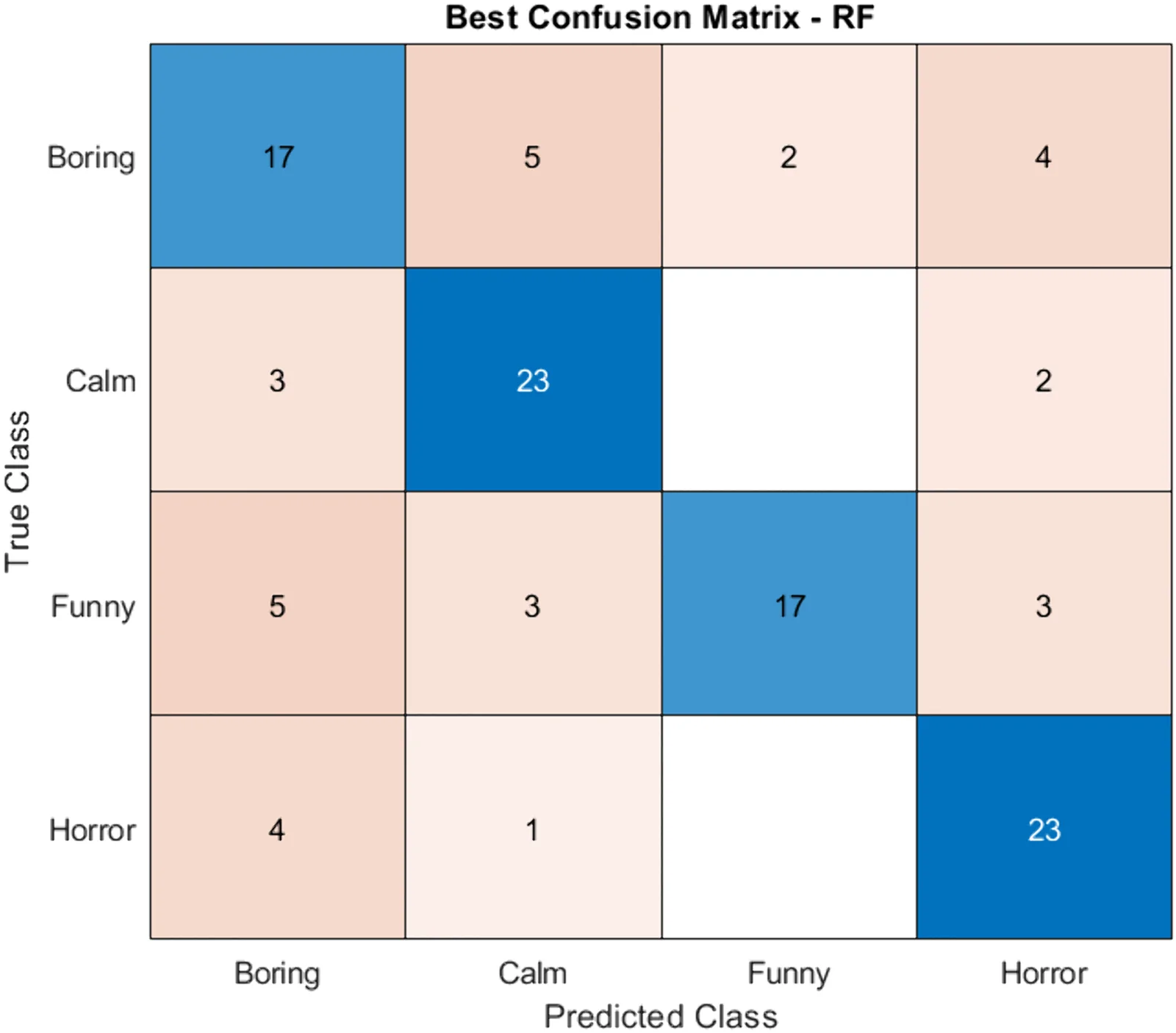
Confusion matrix for RF using EoE – phase features, showing the highest correct classification for the “horror” class.

**Fig 13:**
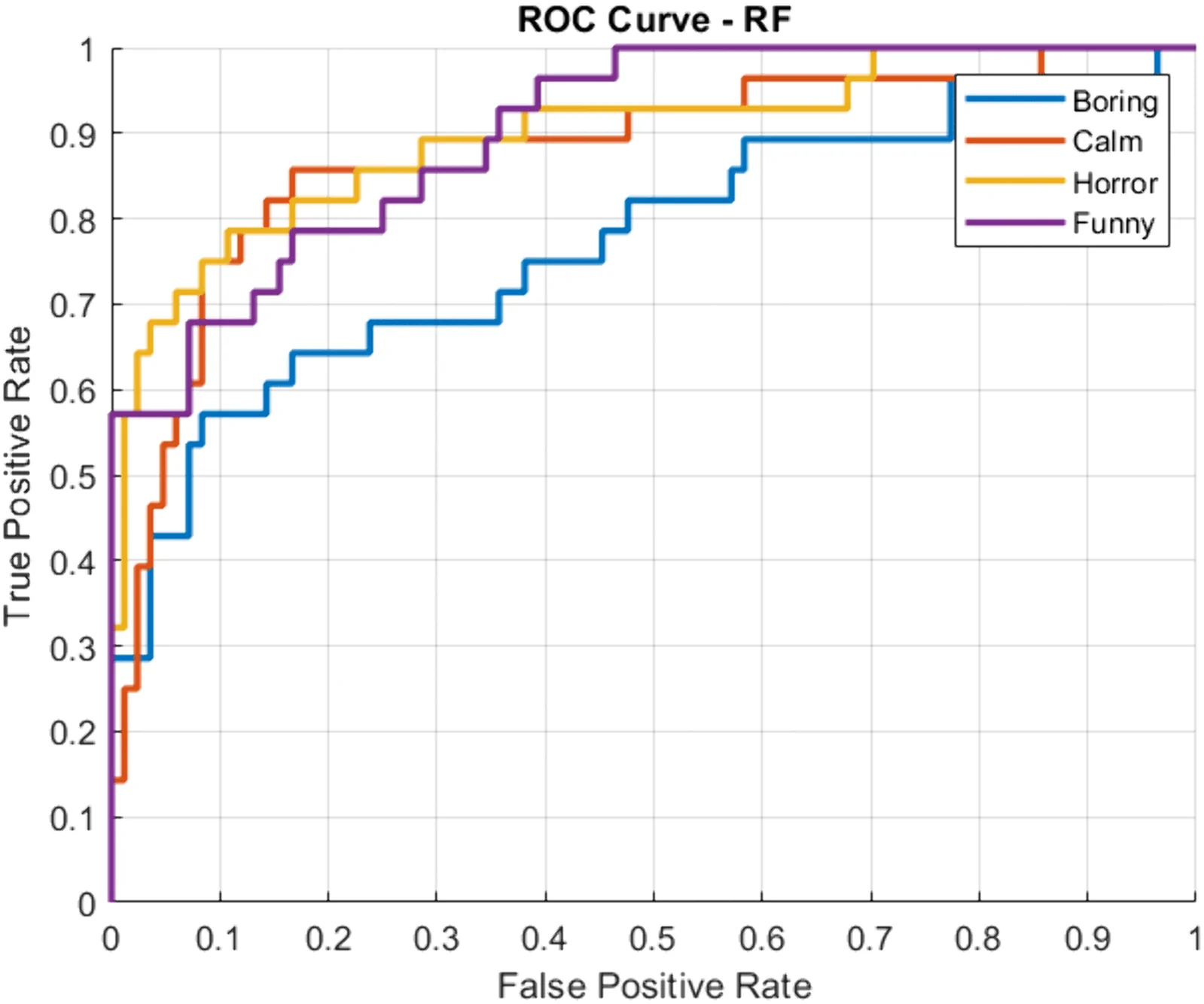
ROC curve for RF using EoE – phase features, showing the highest correct classification for the “horror” class.

**Fig 14:**
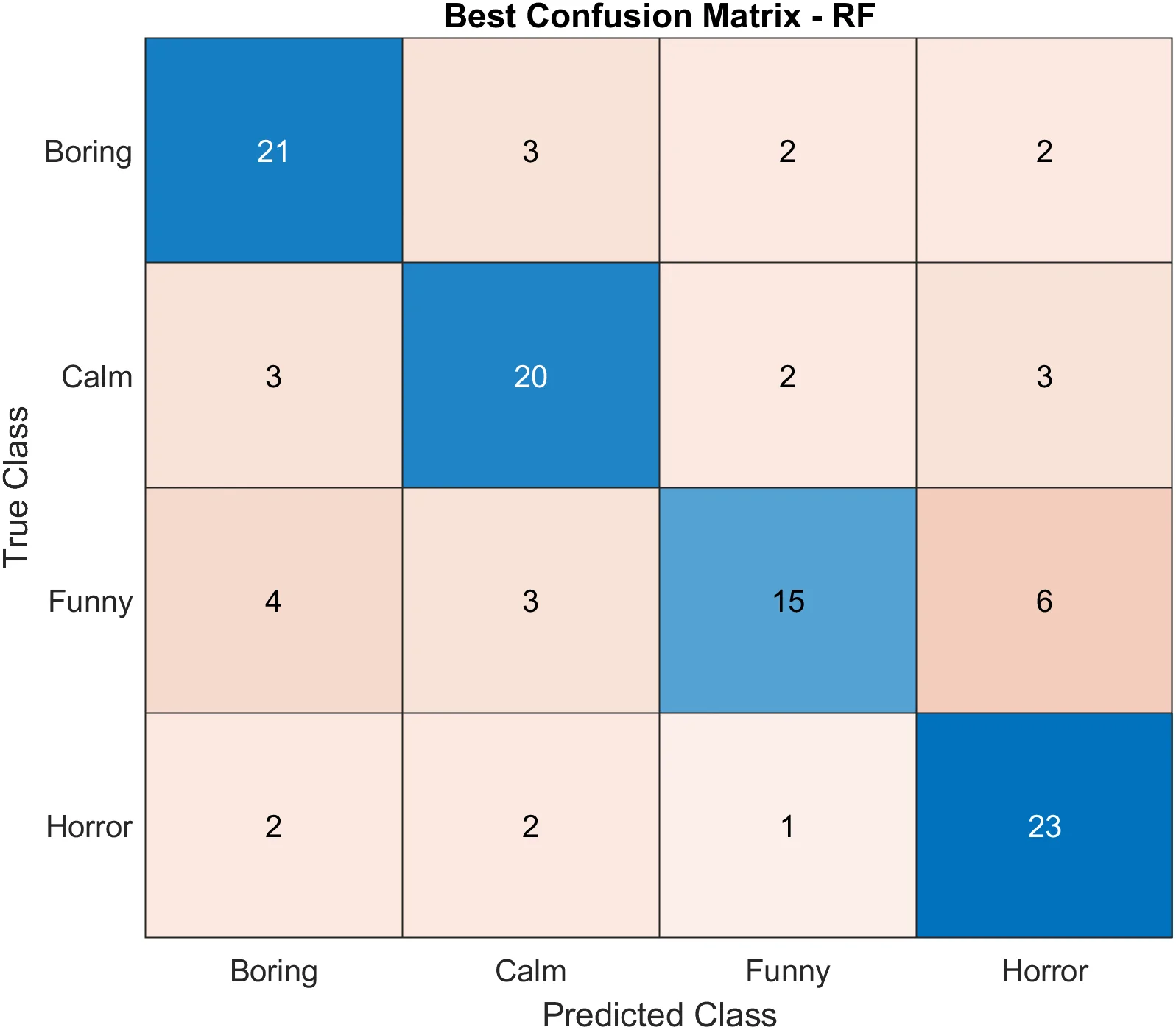
Confusion matrix for PhaseEn features using RF classifier.

**Fig 15:**
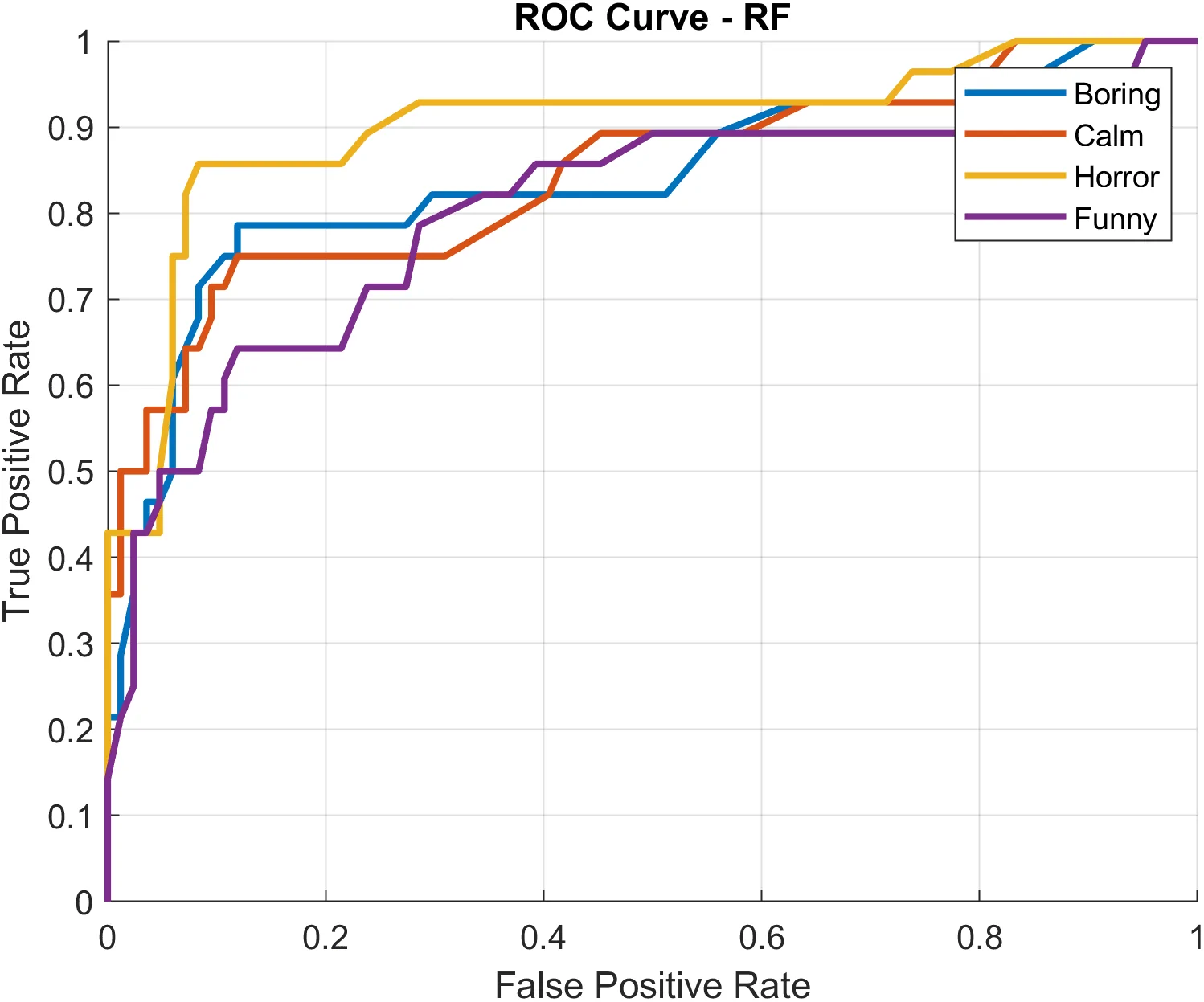
ROC-curve for PhaseEn features using RF classifier.

Importantly, combining PhaseEn with EoE–phase features led to a further improvement in classification performance (Fig 16). As shown in Fig 17, the RF classifier achieved the best overall results using the fused feature set, reaching a final accuracy of 71.7% and demonstrating enhanced class separability, particularly for the previously challenging “Funny” category.

**Fig 16:**
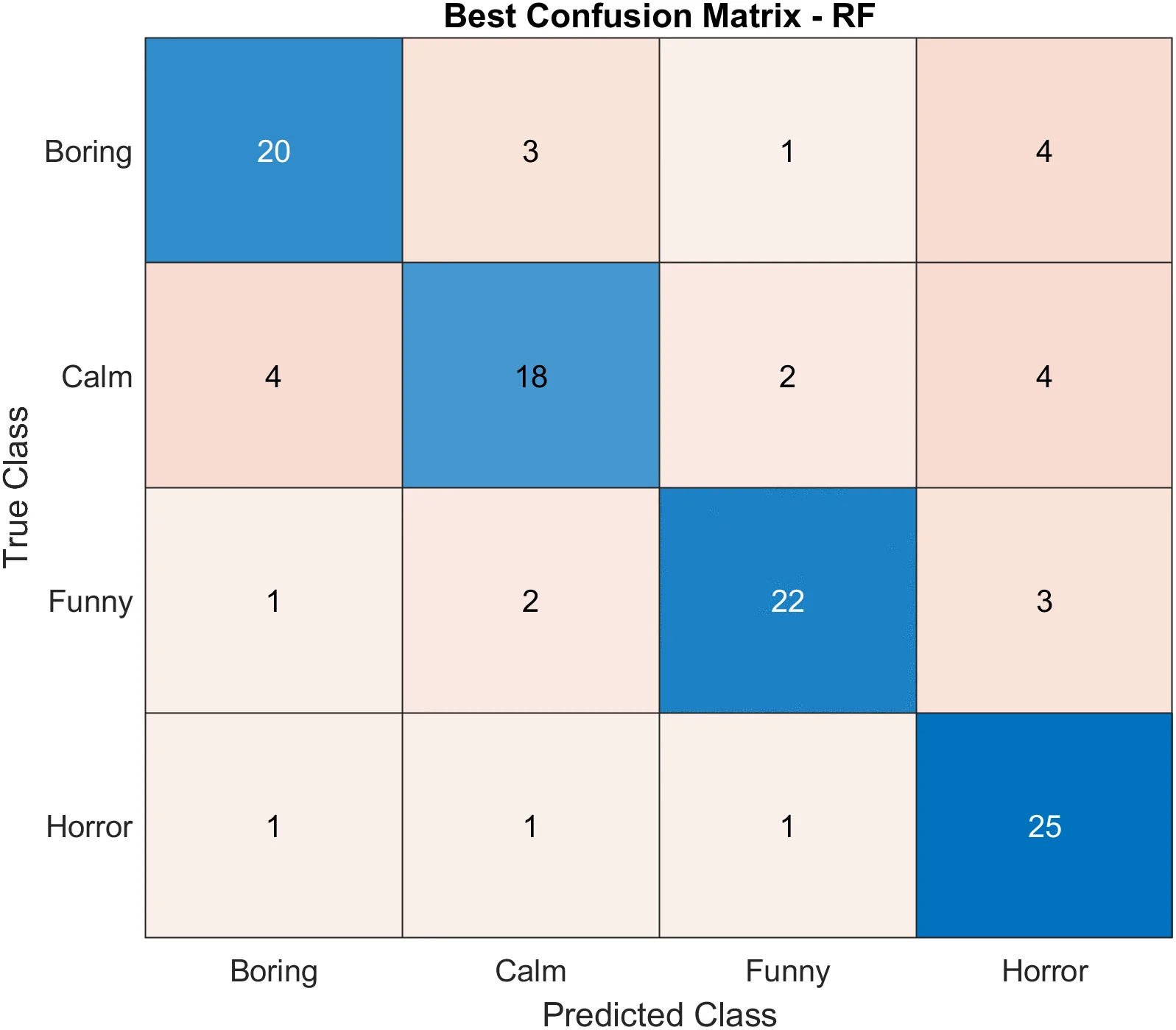
Confusion matrix for PhaseEn + EoE – phase features using RF, showing enhanced resolution for the “Horror” state.

**Fig 17:**
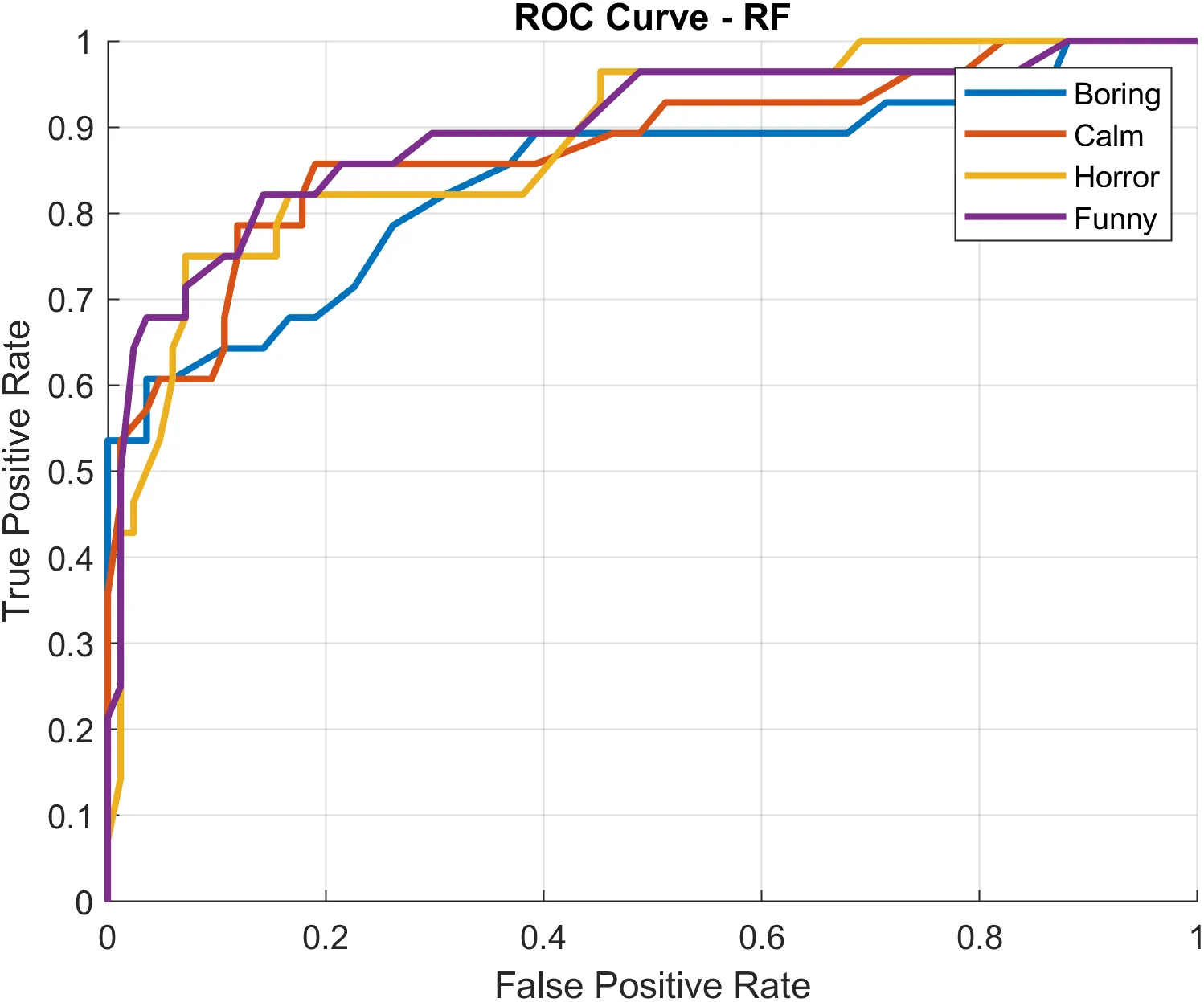
ROC-curve for PhaseEn + EoE – phase features using RF, showing enhanced resolution for the “Horror” state.

In contrast, Shannon-based features resulted in lower overall classification scores (ShanEn achieves accuracy, F1-score, and AUC-ROC values of 0.58.8%, 0.572, and 0.794, respectively, whereas EoE – time yields 35.5%, 0.341, and 0.610). Figs 18–21 present ROC curves and confusion matrices for ShanEn and EoE – time using AdaBoost, revealing reduced separability among emotional classes, particularly in the “Horror” and “Calm” categories.

**Fig 18:**
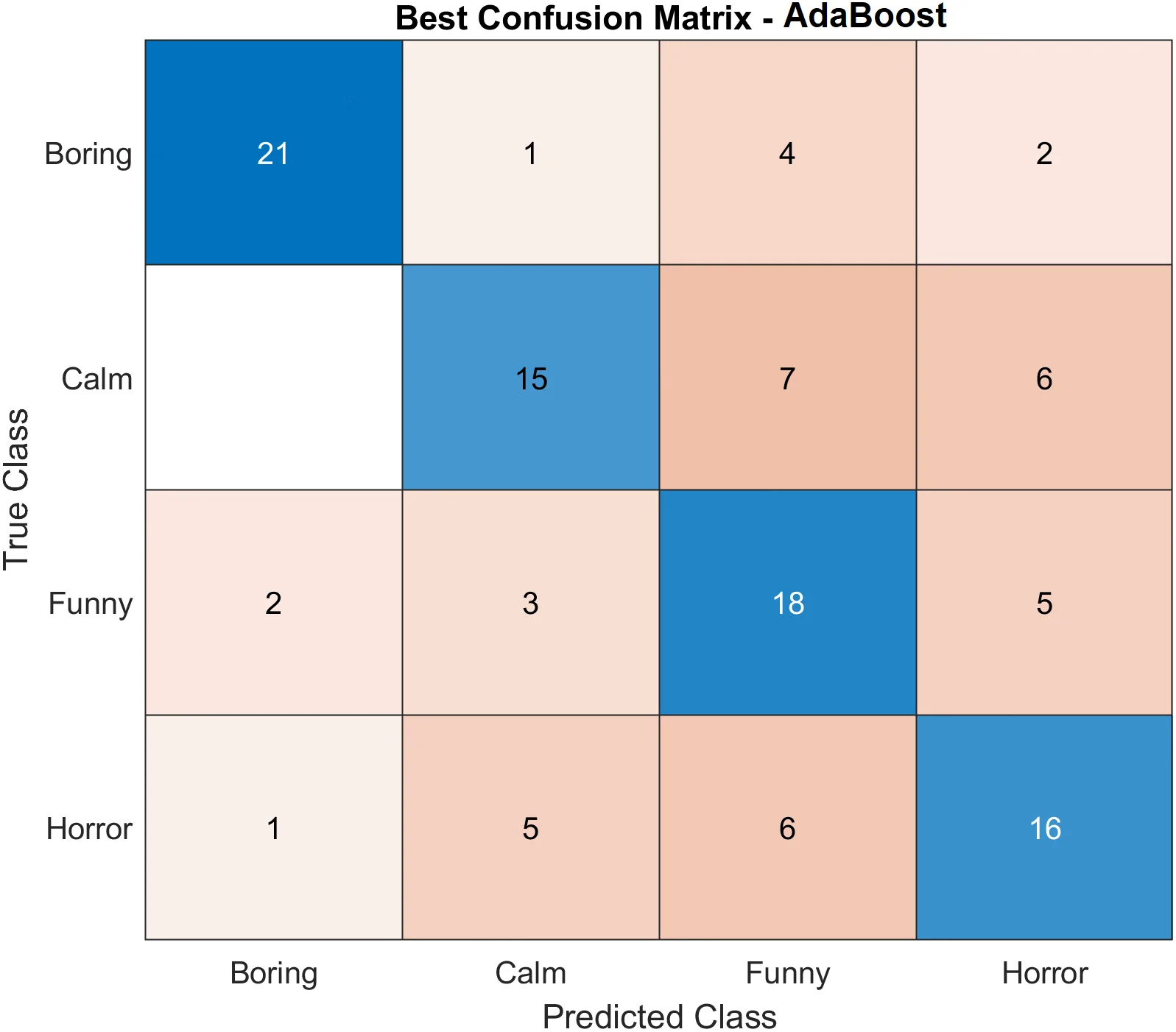
Confusion matrix for the AdaBoost classifier using ShanEn features, showing lower discriminability compared to phase-based features.

**Fig 19:**
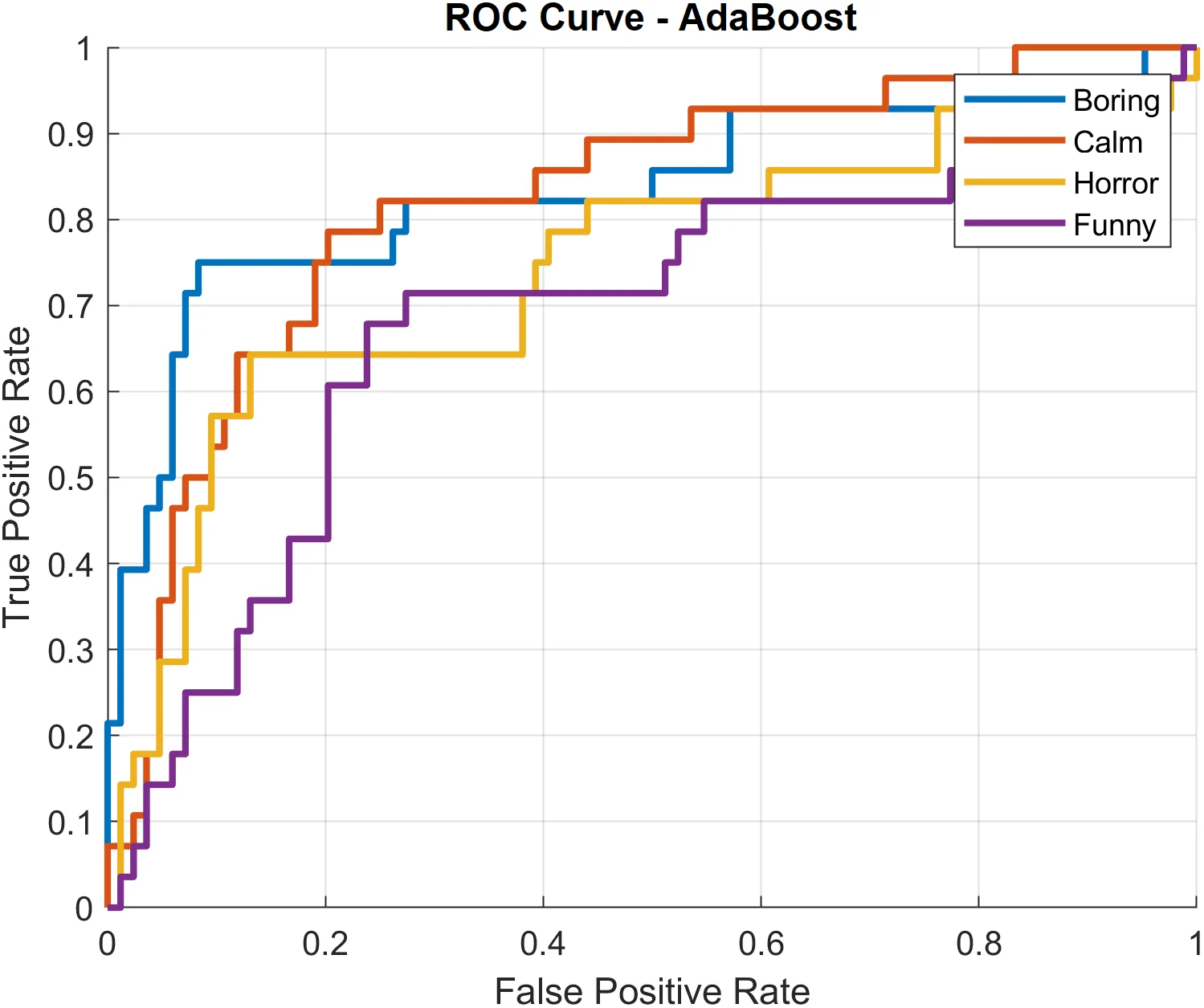
ROC curves for the AdaBoost classifier using ShanEn features, showing lower discriminability compared to phase-based features.

**Fig 20:**
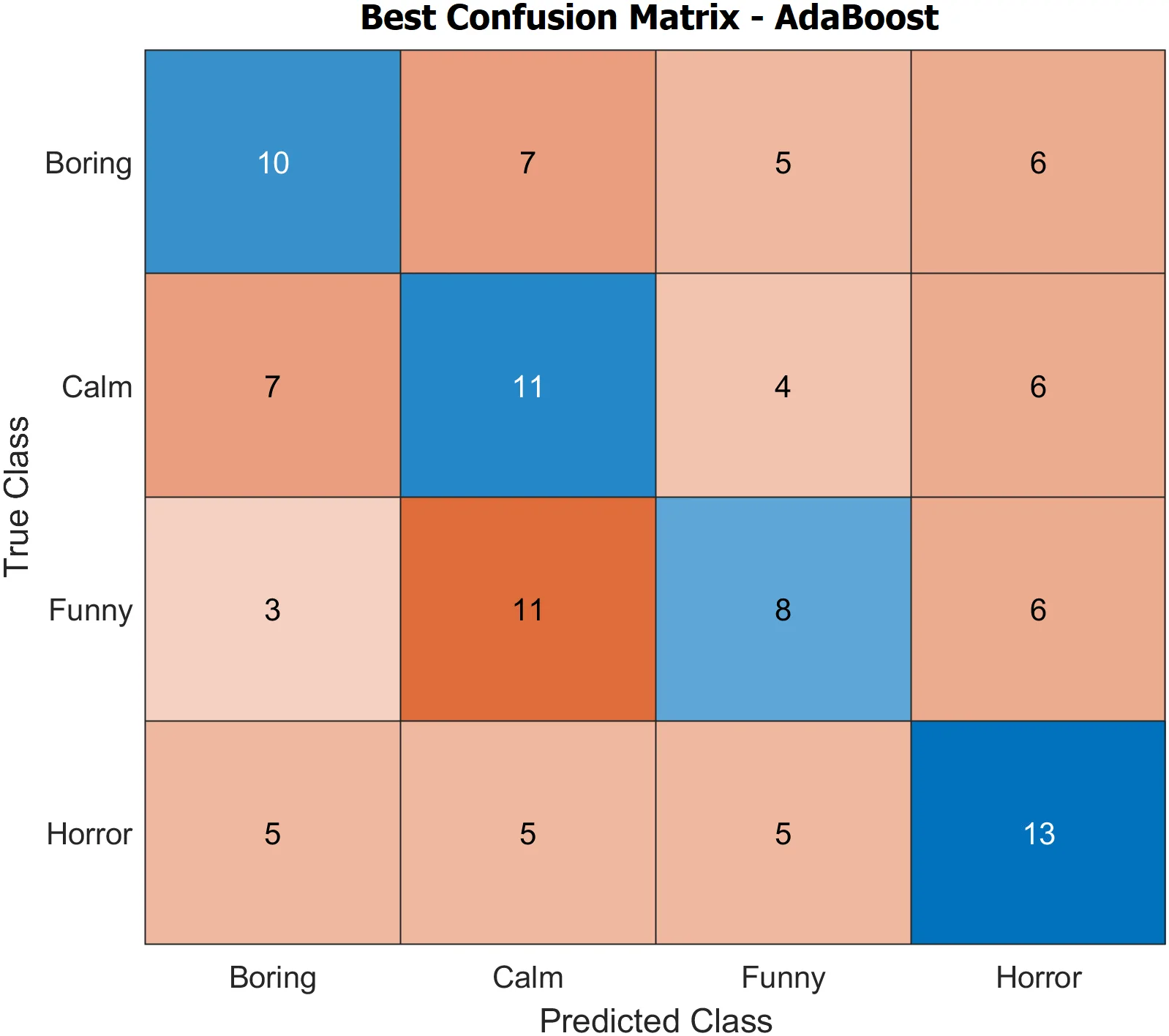
Confusion matrix for the EoE – time features using AdaBoost, showing moderate performance in low-arousal classes.

**Fig 21:**
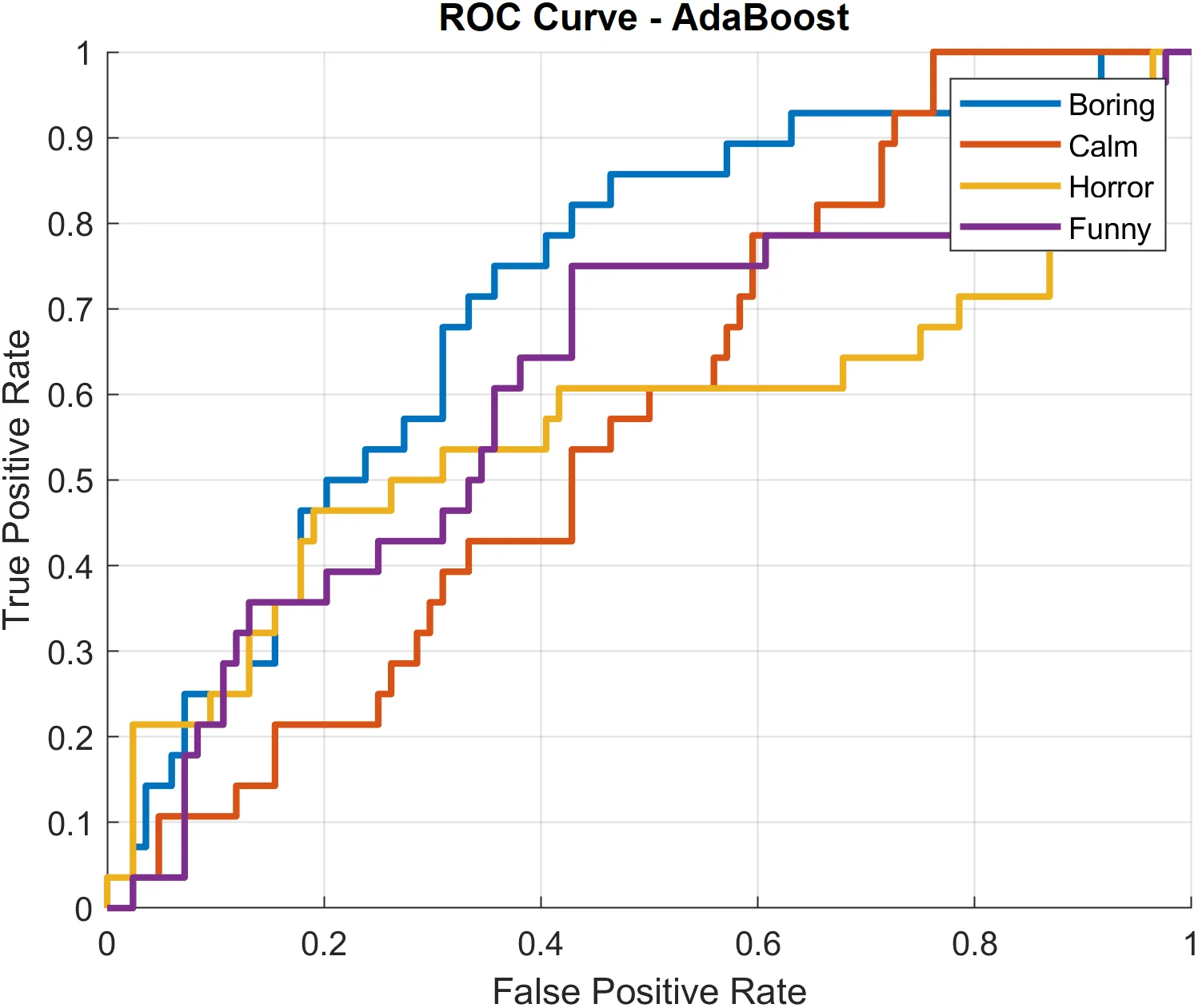
ROC curve for the EoE – time features using AdaBoost, showing moderate performance in low-arousal classes.

Overall, these results confirm that PhaseEn—especially EoE—provides superior discriminative capacity for EEG-based emotion classification. Ensemble classifiers, particularly RF, show consistent performance advantages when paired with robust nonlinear features. The summarized metrics of the top-performing models for each feature set are plotted in Fig 22, showcasing the superiority of a mixture of EoE –phase and PhaseEn in all four metrics: Accuracy, F1-score, AUC-ROC, and AUC-PR.

**Fig 22:**
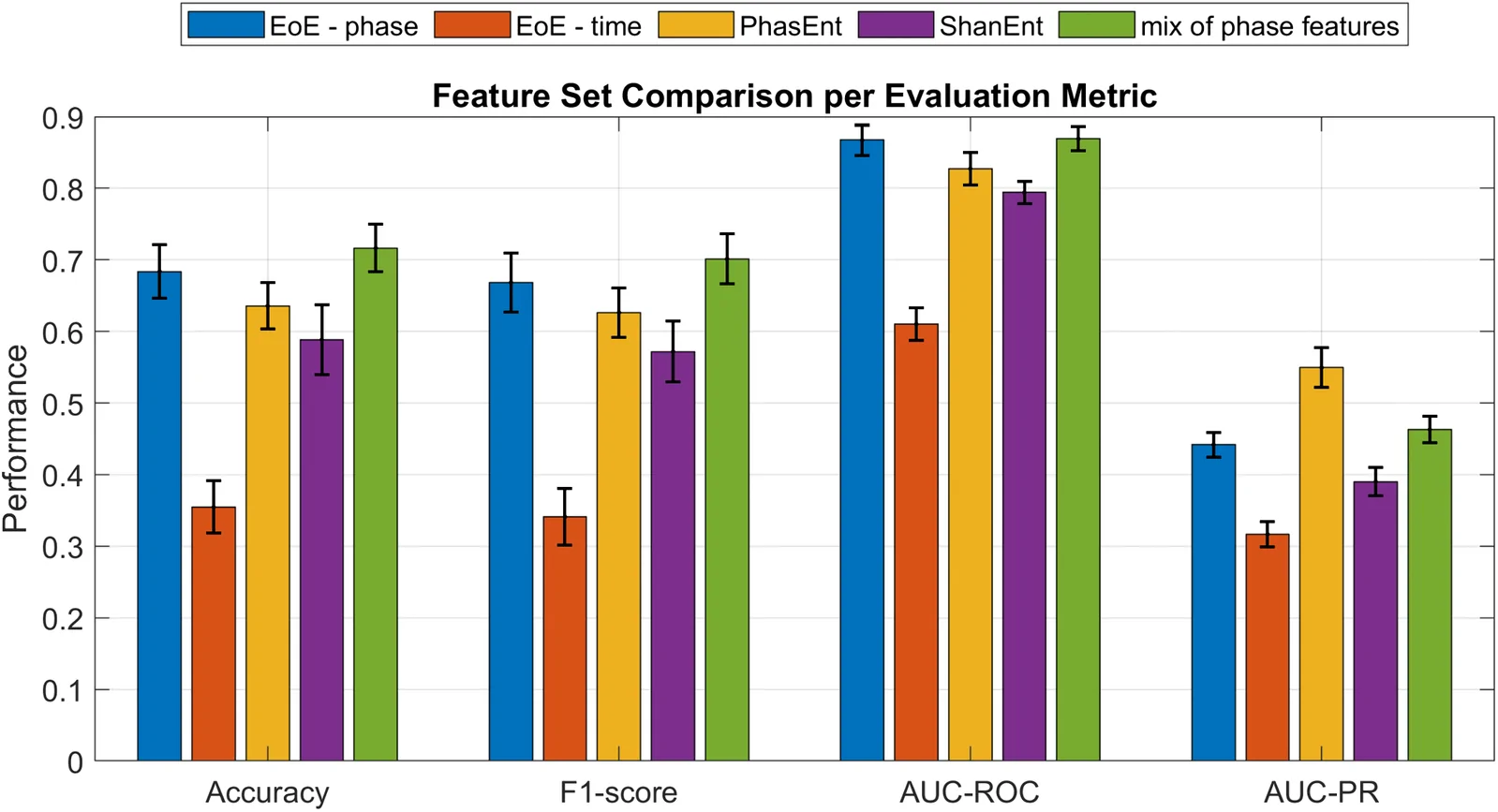
Classification accuracy, F1-score, AUC-PR, and AUC-ROC results for all four feature sets adopted from their best classifiers.

To assess whether the mixed feature set (PhaseEn + EoE-phase) significantly outperforms individual feature sets, we performed pairwise statistical tests on the classification accuracy obtained from 10 independent runs. Normality of the accuracy scores was confirmed using the Lilliefors test (p > 0.05 for all feature sets). Therefore, a one-tailed paired t-test was used for each comparison. The mixed feature set achieved significantly higher accuracy than EoE-phase alone (p = 0.016), PhaseEn alone (p = 0.011), EoE-time (p < 0.001), and Shannon entropy (p < 0.001). Similar significant improvements were observed for F1-score, AUC-ROC, and AUC-PR (all p < 0.05). These results confirm that combining first-order and second-order phase entropy features provides statistically superior emotion recognition performance.

## Discussion

This study presents a comprehensive investigation of emotion-related EEG dynamics through multilevel entropy modeling, with a specific focus on phase-based entropy and its second-order extension, EoE. The overarching aim is to explore how temporal fluctuations in phase information reflect emotion-specific neural dynamics across cortical regions.

We evaluated five feature sets— PhaseEn, EoE – phase, ShanEn, EoE – time, and their combination (PhaseEn + EoE – phase)—using six classifiers (KNN, LDA, SVM, DT, RF, and AdaBoost). The selected baseline methods were chosen to enable a controlled evaluation of the proposed methodological contributions, rather than to maximize the number of competing entropy metrics. The classification results indicate that second-order phase variability contributes meaningful discriminative information for emotion-related neural dynamics among the evaluated feature sets (Accuracy: 71.7%, F1-score: 0.701, AUC-ROC: 0.869). Importantly, EoE-phase outperformed all evaluated baseline feature sets, suggesting that emotional EEG dynamics are more effectively characterized by combining phase information with hierarchical entropy analysis than by conventional entropy representations alone.

This comparative framework was intentionally designed to isolate the individual contributions of the two methodological components introduced in this work: phase representation and hierarchical entropy analysis. Accordingly, Shannon entropy, Phase entropy, and EoE-time were selected because they directly represent these methodological components and allow their individual effects to be evaluated without introducing additional algorithmic differences. Therefore, the objective of this study was not to provide an exhaustive benchmark of all entropy measures, but rather to determine whether incorporating phase information and second-order entropy analysis provides additional discriminative power for emotion-related EEG signals. Although broader comparisons with other nonlinear entropy measures, such as Sample Entropy and Permutation Entropy, would provide valuable complementary benchmarks, such comparisons address a different research objective and are therefore left for future work.

PhaseEn acts as a complementary low-level descriptor with limited statistical separability at the single-channel level, but its contribution becomes more pronounced in multivariate classification settings where nonlinear interactions across channels are exploited by machine learning models.

Unlike PhaseEn, EoE-phase explicitly captures higher-order temporal variability, providing a richer representation of dynamic neural fluctuations associated with emotional processing. This improved discriminability may be associated with the fact that neural phase reflects the timing of oscillatory cycles underlying functional integration across brain regions. Emotions, being inherently dynamic and multimodal, are not strictly localized to one region but involve distributed synchronization patterns.

Topographical analysis showed that five channels—T7, T8, AF4, P8 and O2—consistently contributed to emotion differentiation based on EoE – phase features. These spatial patterns carry strong neurophysiological implications:

- T7 and T8 (Bilateral Temporal Lobes): These regions are involved in auditory processing, semantic appraisal, and affective interpretation of scenes. Their increased entropy variability during the horror condition suggests active sensory-emotional integration or altered engagement. Temporal regions also support memory-related aspects of emotional processing, particularly in fear-based scenarios [37].
- AF4 (Right Frontal Pole): This region is central to emotional regulation, executive control, and approach/avoidance behavior [38]. Asymmetrical activation in the frontal pole—especially right-dominant activity—is associated with heightened emotional salience and arousal. High or low EoE here may reflect top-down modulation of emotional experience [39].
- P8 (Right Parietal Cortex): The right parietal region plays a key role in attentional allocation, multisensory integration, and orienting to emotionally salient stimuli. Variability in entropy at P8 may reflect dynamic shifts in attentional engagement during affective processing [40].
- O2 (Right Occipital Cortex): Beyond its primary role in visual perception, O2 has been implicated in emotionally modulated visual attention and imagery. Elevated entropy dynamics here during boredom or calm may indicate shifting visual engagement or mental imagery under low arousal [41,42].

The observed channel-specific modulation patterns of EoE-phase across emotional conditions suggest a potentially distributed and functionally differentiated contribution of cortical regions to emotion-related neural dynamics. Overall, these observations should be interpreted cautiously as they may suggest a network-level differentiation between frontal regulatory systems and temporo-occipital perceptual–affective regions. Rather than implying direct causal mechanisms, they highlight the potential of EoE-phase to capture distributed and emotion-dependent variability in neural dynamics.

It is important to consider what cognitive processes the classifiers may have captured. Based on our results and the nature of the GAMEEMO dataset, we hypothesize that the classifiers leverage a combination of at least three factors. First, perceptual differences between the four video stimuli (funny, boring, horror, calm) likely contribute to the classification, as each stimulus has distinct audiovisual features [43]. However, the fact that EoE-phase features were derived from EEG phase dynamics—rather than low-level sensory evoked potentials—suggests that the classifiers are not merely decoding sensory input [18]. Stable individual differences in baseline EEG characteristics could also influence classification, although our subject-independent cross-validation reduces the risk of overfitting to specific participants. Disentangling these factors remains a challenge for current affective computing studies, and future research using process-oriented tasks (e.g., with explicit regulation instructions or continuous emotion ratings) would help clarify the specific contributions of perception, regulation, and trait-like variability [19].

The phase of neural oscillations encodes timing relationships between brain regions, reflecting functional connectivity [44,45]. In the context of emotion, phase-based analyses capture coordinated activity patterns that underlie the synchronization of attention, memory, and valence/arousal processing [46]. EoE-phase consistently showed higher discriminative capability compared to Shannon entropy-based features across classifiers, indicating that second-order variability in phase dynamics may provide complementary information to traditional entropy measures. This apparent discrepancy between statistical significance and classification performance arises from the difference between univariate statistical testing and multivariate nonlinear classification, where feature interactions play a critical role. These findings are consistent with prior literature emphasizing the importance of phase–locking and desynchronization in emotional and cognitive processing [47], as shown in the works of Klimesch [48] and Başar et al [49].

These findings collectively suggest that EoE captures how different brain areas dynamically reconfigure their temporal activity in response to varying emotional demands. Rather than viewing emotional EEG as static signatures, our results support a view of emotion as a transient, regionally distributed process governed by fluctuating neural synchrony.

Furthermore, classifier comparisons revealed that nonlinear models (RF, AdaBoost) tend to be more effective at capturing the complex interactions within high-dimensional phase entropy features, compared to linear models (LDA) or distance-based models (KNN). This suggests that emotion-related EEG features exhibit nonlinear separability that benefits from ensemble-based decision boundaries. The current findings should be interpreted as exploratory rather than definitive evidence of causal neural mechanisms.

In summary, this work emphasizes three key findings: (1) EoE – phase is a sensitive marker of dynamic emotional states, (2) cortical contributions to emotion are spatially heterogeneous and vary in temporal stability, and (3) second-order entropy metrics provide a novel means to capture both the content and variability of emotional processing across time and space. Overall, these results support a network-oriented interpretation of emotion, in which temporal variability of neural synchronization plays a central role.

## Conclusion

This study introduced and validated a novel approach for EEG-based emotion recognition using EoE applied to phase information. Through comprehensive statistical and machine learning analyses, we demonstrated that EoE – phase features offer superior emotional discriminability across EEG channels and emotional categories. From just 14 scalp electrodes, we successfully identified five emotion-sensitive brain regions—T7, T8, AF4, P8, and O2—highlighting the feasibility of compact, wearable EEG systems for real-world affective computing. These regions are consistent with established literature on temporal, frontal, and occipital involvement in emotional perception and regulation. Our key innovation lies in treating entropy as a time-evolving signal, rather than a static summary statistic. The EoE framework effectively captures second-order complexity, allowing dynamic variations in EEG activity to inform emotional classification. Comparative results showed that EoE – phase clearly outperforms both raw PhaseEn and conventional ShanEn— in terms of statistical significance and classifier accuracy. Classification results using RF reached 71.7% accuracy, 0.701 F1 – score and 0.869 AUC for four-class emotion recognition, outperforming alternative feature-model combinations. These findings suggest that phase dynamics and their temporal structure are central to understanding and decoding emotional brain states.

Despite promising results, this work has limitations. The GAMEEMO dataset includes only four discrete emotional states and a relatively homogeneous subject group. Future research should expand to continuous emotion models (valence-arousal space) and larger, more diverse populations. Moreover, while EoE – phase is effective, future studies could explore hybrid entropy models (e.g., combining EoE with PermEn or multivariate EoE) and their integration into deep learning pipelines for end-to-end optimization. Another avenue is to investigate phase synchronization and EoE across network-level EEG connectivity to assess how emotional coordination arises across brain regions. Ultimately, the findings of this study establish EoE – phase as a powerful and interpretable marker for EEG-based emotion recognition, paving the way for next-generation affective brain-computer interfaces in gaming, mental health, and human-computer interaction.

## Supporting information

S1 Table Pipeline-level parameter sensitivity: Effect of segment duration, N, and M on the number of significant EoE channels. (DOCX)

S2 Table Normality (Kolmogorov–Smirnov) and statistical analysis summary per channel and feature. (DOCX)
