# Supplementary material for: EEG emotion recognition via multilevel entropy analysis: The power of entropy of entropy on phase dynamics
Source: PLoS One. 2026 Oct 5;21(10):e0329598. doi: 10.1371/journal.pone.0329598 (PMC13637881; doi:10.1371/journal.pone.0329598)
Supplement: Supplementary file 1 [file pone.0329598.s001.docx]

**S1 Table– Pipeline-level parameter sensitivity: effect of segment duration, N, and M on the number of significant EoE channels**

| **Segment (s)** | **N** | **M** | **Number of significant channels**  **(EoE)** |
| --- | --- | --- | --- |
| 0.5 | 12 | 5 | 2 |
| 0.5 | 12 | 8 | 3 |
| 0.5 | 8 | 5 | 1 |
| 0.5 | 8 | 8 | 3 |
| 1 | 12 | 5 | 5 |
| 1 | 12 | 8 | 3 |
| 1 | 8 | 5 | 0 |
| 1 | 8 | 8 | 2 |
| 2 | 12 | 5 | 4 |
| 2 | 12 | 8 | 1 |
| 2 | 8 | 5 | 3 |
| 2 | 8 | 8 | 2 |
