# Supplementary material for: EEG emotion recognition via multilevel entropy analysis: The power of entropy of entropy on phase dynamics
Source: PLoS One. 2026 Oct 5;21(10):e0329598. doi: 10.1371/journal.pone.0329598 (PMC13637881; doi:10.1371/journal.pone.0329598)
Supplement: Supplementary file 2 [file pone.0329598.s002.docx]

**Table S2. Normality (Kolmogorov–Smirnov) and statistical analysis summary per channel and feature**

| **Feature** | **Channel** | **KS test (p-value)** | **Distribution** | **Main test used** | **Main test p-value** |
| --- | --- | --- | --- | --- | --- |
| PhaseEn | AF3 | 0.503446648 | Normal | ANOVA -> Tukey | 0.807615498 |
| EoE | AF3 | 0.874525545 | Normal | ANOVA -> Tukey | 0.13281438 |
| PhaseEn | AF4 | 0.05069195 | Normal | ANOVA -> Tukey | 0.729421505 |
| **EoE** | **AF4** | **0.544116518** | **Normal** | **ANOVA -> Tukey** | **0.010610969** |
| PhaseEn | F3 | 6.04297E-05 | Non-normal | Kruskal-Wallis -> Dunn | 0.25339845 |
| EoE | F3 | 0.280327527 | Normal | ANOVA -> Tukey | 0.238414387 |
| **PhaseEn** | **F4** | **0.001910407** | **Non-normal** | **Kruskal-Wallis -> Dunn** | **0.02811954** |
| EoE | F4 | 0.054695503 | Normal | ANOVA -> Tukey | 0.15237868 |
| PhaseEn | F7 | 0.000709812 | Non-normal | Kruskal-Wallis -> Dunn | 0.680989169 |
| EoE | F7 | 0.075060637 | Normal | ANOVA -> Tukey | 0.496639659 |
| PhaseEn | F8 | 8.8665E-06 | Non-normal | Kruskal-Wallis -> Dunn | 0.121756381 |
| EoE | F8 | 0.65058022 | Normal | ANOVA -> Tukey | 0.362850569 |
| PhaseEn | FC5 | 2.06652E-06 | Non-normal | Kruskal-Wallis -> Dunn | 0.697497585 |
| EoE | FC5 | 0.682610079 | Normal | ANOVA -> Tukey | 0.092005698 |
| PhaseEn | FC6 | 9.09082E-08 | Non-normal | Kruskal-Wallis -> Dunn | 0.252497233 |
| EoE | FC6 | 0.013136951 | Non-normal | Kruskal-Wallis -> Dunn | 0.213136787 |
| PhaseEn | O1 | 5.44026E-06 | Non-normal | Kruskal-Wallis -> Dunn | 0.912594146 |
| EoE | O1 | 0.347062363 | Normal | ANOVA -> Tukey | 0.592050362 |
| PhaseEn | O2 | 0.401504295 | Normal | ANOVA -> Tukey | 0.708497616 |
| **EoE** | **O2** | **0.188720032** | **Normal** | **ANOVA -> Tukey** | **0.026968357** |
| PhaseEn | P7 | 0.005572872 | Non-normal | Kruskal-Wallis -> Dunn | 0.644125599 |
| EoE | P7 | 0.265536099 | Normal | ANOVA -> Tukey | 0.165284032 |
| PhaseEn | P8 | 0.164349733 | Normal | ANOVA -> Tukey | 0.678539551 |
| **EoE** | **P8** | **0.421100401** | **Normal** | **ANOVA -> Tukey** | **0.005038948** |
| PhaseEn | T7 | 0.000139929 | Non-normal | Kruskal-Wallis -> Dunn | 0.312604395 |
| **EoE** | **T7** | **0.668103174** | **Normal** | **ANOVA -> Tukey** | **0.000863575** |
| PhaseEn | T8 | 5.5049E-11 | Non-normal | Kruskal-Wallis -> Dunn | 0.076926657 |
| **EoE** | **T8** | **0.070625472** | **Normal** | **ANOVA -> Tukey** | **0.011003837** |
